# Sustained NFκB inhibition improves insulin sensitivity but is detrimental to muscle health

**DOI:** 10.1111/acel.12613

**Published:** 2017-05-29

**Authors:** Ning Zhang, Joseph M. Valentine, You Zhou, Mengyao E. Li, Yiqiang Zhang, Arunabh Bhattacharya, Michael E. Walsh, Katherine E. Fischer, Steven N. Austad, Pawel Osmulski, Maria Gaczynska, Steven E. Shoelson, Holly Van Remmen, Hung I. Chen, Yidong Chen, Hanyu Liang, Nicolas Musi

**Affiliations:** ^1^ Barshop Institute for Longevity and Aging Studies University of Texas Health Science Center at San Antonio 15355 Lambda Drive San Antonio TX 78245 USA; ^2^ Joslin Diabetes Center 1 Joslin Place Boston MA 02215 USA; ^3^ Greehey Children's Cancer Research Institute 8403 Floyd Curl Dr San Antonio TX 78229 USA; ^4^ Department of Epidemiology and Biostatistics University of Texas Health Science Center at San Antonio 7703 Floyd Curl Dr San Antonio TX 78229 USA; ^5^ San Antonio Geriatric Research, Education and Clinical Center South Texas Veterans Health Care System 7400 Merton Minter San Antonio TX 78229 USA; ^6^ Present address: University of Alabama at Birmingham Birmingham AL USA; ^7^ Present address: The Oklahoma Medical Research Foundation Oklahoma OK USA

**Keywords:** aging, insulin resistance, NFκB, sarcopenia, skeletal muscle

## Abstract

Older adults universally suffer from sarcopenia and approximately 60–70% are diabetic or prediabetic. Nonetheless, the mechanisms underlying these aging‐related metabolic disorders are unknown. NFκB has been implicated in the pathogenesis of several aging‐related pathologies including sarcopenia and type 2 diabetes and has been proposed as a target against them. NFκB also is thought to mediate muscle wasting seen with disuse, denervation, and some systemic diseases (*e.g*., cancer, sepsis). We tested the hypothesis that lifelong inhibition of the classical NFκB pathway would protect against aging‐related sarcopenia and insulin resistance. Aged mice with muscle‐specific overexpression of a super‐repressor IκBα mutant (MISR) were protected from insulin resistance. However, MISR mice were not protected from sarcopenia; to the contrary, these mice had decreases in muscle mass and strength compared to wild‐type mice. In MISR mice, NFκB suppression also led to an increase in proteasome activity and alterations in several genes and pathways involved in muscle growth and atrophy (*e.g*., myostatin). We conclude that the mechanism behind aging‐induced sarcopenia is NFκB independent and differs from muscle wasting due to pathologic conditions. Our findings also indicate that, while suppressing NFκB improves insulin sensitivity in aged mice, this transcription factor is important for normal muscle mass maintenance and its sustained inhibition is detrimental to muscle function.

## Introduction

Aging is characterized by a deterioration in glucose homeostasis and the progressive loss of muscle mass and function. However, the molecular basis for insulin resistance and sarcopenia of aging is unknown. The transcription factor NFκB is a key regulator of inflammatory responses. Accumulating evidence suggests that the NFκB pathway may be involved in the aging process and in the pathogenesis of various aging‐related pathologies. For example, upregulation of the NFκB pathway in aged animals and older humans has been demonstrated in multiple tissues such as skeletal muscle, liver, kidney, heart, and gastric mucosa (Helenius *et al*., 1996; Korhonen *et al*., 1997; Walter & Sierra, 1998; Xiao & Majumdar, 2000; Ghosh *et al*., 2015). In line with these findings, a global gene expression microarray analysis of various human and mouse tissues determined that the NFκB motif was the motif most strongly associated with aging (Adler *et al*., 2007).

NFκB is upregulated in muscle of both aged rodents (Phillips & Leeuwenburgh, 2005) and human subjects (Buford *et al*., 2010; Thalacker‐Mercer *et al*., 2010; Ghosh *et al*., 2015), and the NFκB pathway has been implicated in the pathogenesis of insulin resistance and type 2 diabetes (Yuan *et al*., 2001). Therefore, the NFκB pathway may be involved in the glucose metabolism abnormalities that occur during aging. NFκB also is thought to play a key role in the muscle wasting seen with cancer, muscle disuse, and denervation (Cai *et al*., 2004; Judge *et al*., 2007; Van Gammeren *et al*., 2009; Reed *et al*., 2011). Thus, NFκB may also mediate the muscle loss characteristic of the aging process. Despite the evidence indicating that NFκB may be involved in the glucose metabolism abnormalities and muscle wasting seen with certain pathologic conditions (*e.g*., type 2 diabetes, cancer, denervation, and disuse), it is not known whether NFκB is a link between the pathogenesis of insulin resistance and the muscle atrophy of aging. In this study, we tested the hypothesis that muscle‐specific NFκB inactivation would protect against aging‐induced insulin resistance and sarcopenia. If positive, these findings would demonstrate a role for NFκB on the pathophysiology of these metabolic disorders that frequently affect older adults. Such results also would provide proof‐of‐concept that interventions that target NFκB could be effective in the prevention and treatment of aging‐related metabolic conditions.

## Results

### Animal model

We studied C57BL/6 mice with muscle‐specific overexpression of an IκBα super‐repressor mutant (MISR) and wild‐type (WT) littermates. Generation of these mice and genotyping procedures were described previously (Cai *et al*., 2004). This is a well‐characterized model for studies of suppressed NFκB activity, which carries an IκBα transgene (S32A/S36A) that functions as a potent dominant negative inhibitor of the classical NFκB pathway, because mutating serines 32 and 36 for alanine prevents phosphorylation and dissociation of IκBα from NFκB p50/65. Expression of the transgene in skeletal muscle is driven by a muscle creatine kinase (MCK) promoter. Mice were genotyped by PCR, and overexpression of IκBα (*nfkbia*) gene in muscle was confirmed by RNA sequencing (Fig. S1, Supporting information). Mice were housed in an animal room maintained at 23 °C with a 12‐h light/12‐h dark cycle and were provided standard laboratory chow and water *ad libitum*. We studied male and female mice of different ages; the ages and sexes of the mice are described in each experiment.

### Muscle‐specific NFκB inhibition protects against aging‐induced insulin resistance

**Figure 1:**
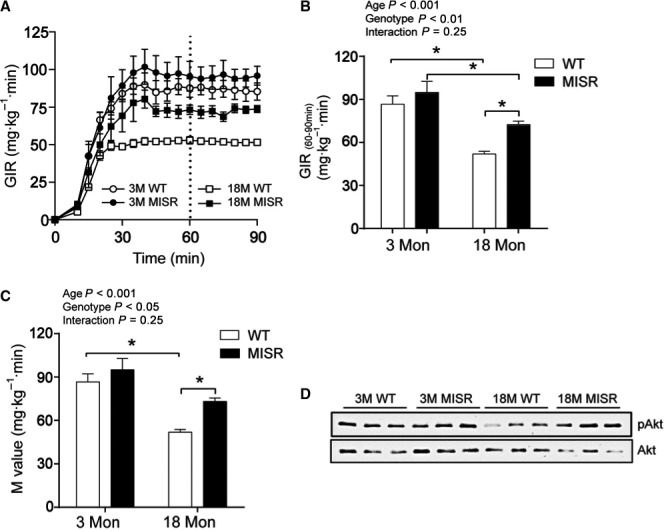
Improved insulin action in MISR mice. Glucose infusion rate (GIR) required to maintain euglycemia and M value in 3‐ and 18‐month‐old male mice. (A) GIR during the clamp. (B) Mean GIR during the last 30 min of the clamp. (C) M value. *n *= 4–5 per group. *P <* *0.05 by two‐way ANOVA followed by Tukey's post hoc test. All data are means ± SE. (D) Akt Ser473 phosphorylation assessed by Western blotting in quadriceps muscle collected at the end of the insulin clamp. Representative blots are shown for each group.

Intraperitoneal glucose tolerance testing (GTT) was performed in male and female mice of various ages (1–3 months, 12–13 months, and 20–22 months). Blood glucose levels during the GTT were similar in male WT and MISR in all age groups (Fig. S2). In 1‐ to 3‐ and 20‐ to 22‐month‐old female MISR mice, plasma glucose was modestly reduced compared with WT. A hyperinsulinemic euglycemic clamp procedure also was performed because it is more precise and sensitive than the GTT assay for quantitation of insulin‐mediated glucose disposal in the periphery (which mainly reflects muscle glucose disposal). Clamp studies were performed in 3‐ and 18‐month‐old male mice. Both the glucose infusion rate (GIR) required to maintain euglycemia (Fig. 1A,B) and the M value (Fig. 1C) were lower in 18‐month‐old WT mice than in 3‐month‐old WT mice by 40%. However, 18‐month‐old MISR mice had increases in both GIR and M compared with aged‐matched WT mice by 29%, indicating improved insulin sensitivity. Consistent with this finding, Akt phosphorylation in muscle tissue harvested at the end of the insulin clamp was higher in 18‐month‐old MISR mice vs. age‐matched WT mice (Fig. 1D). Endogenous glucose production was fully suppressed in all groups during insulin infusion (not shown). Blood glucose and plasma insulin concentrations during the clamp were similar between groups (Fig. S3). In the basal (noninsulin stimulated) state, Akt phosphorylation was not different between WT and MISR mice in any age group (Fig. S4).

Indirect calorimetry was performed in 3‐, 13‐, and 35‐month‐old male mice to assess the effects of aging and NFκB suppression on whole‐body substrate utilization and energy expenditure. In both genotypes, aging led to modest reductions in oxygen consumption, carbon dioxide production, and resting metabolic rate (Fig. S5). The respiratory quotient also decreased in WT and MISR mice with aging, suggestive of more reliance on lipid oxidation as age advanced. There were no significant differences between genotypes in any of these metabolic parameters. Spontaneous activity during light and dark phases also tended to decrease with age in both genotypes, and aged MISR mice seemed to be less active in the dark phase compared to aged WT mice, although this difference did not reach statistical significance (Fig. S5). Overall, aging led to a subtle hypometabolic state in both genotypes, and NFκB suppression in muscle did not notably affect whole‐body substrate utilization.

### Altered content of lipid metabolites and carnitine in muscle from aged and MISR mice

**Figure 2:**
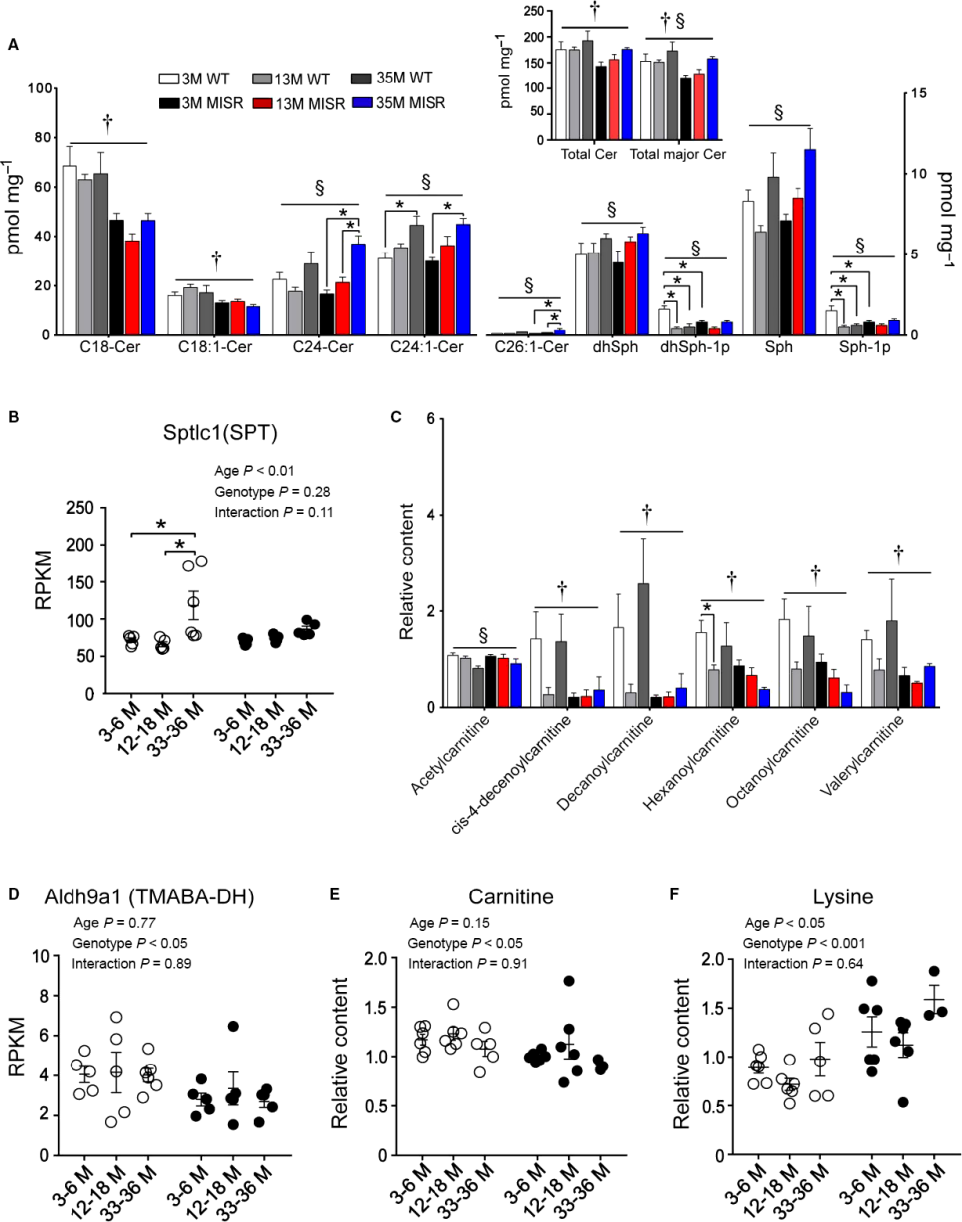
Ceramide and acylcarnitine content in muscles from MISR mice. (A) Ceramide and sphingolipid content in quadriceps muscle.. *n* = 3–6 per group. (B) mRNA levels of *sptlc1* (SPT) in WT (white) and MISR (black) mice. SPT catalyzes the rate‐limiting step in the *de novo* synthesis of sphingolipids. For B, *n *= 4–6 per group. (C) Acylcarnitine content in quadriceps. *n *= 3–6 per group. (D) mRNA levels of the carnitine biosynthetic enzyme *aldh9a1* (TMABA‐DH). *n *= 5–6 per group. (E) Carnitine muscle content. (F) Muscle content of the precursor lysine. For panels E and F, *n *= 3–6 per group. †Genotype effect *P *<* *0.05 by two‐way ANOVA; §Age effect *P *<* *0.05 by two‐way ANOVA; *P <* *0.05 by Tukey's post hoc test. All data are means ± SE.

Muscle content of ceramide, acylcarnitine, and diacylglycerol species was measured in mice from different age groups (3, 13, and 35 months old) as aging is associated with increased intramyocellular lipid accumulation (Petersen *et al*., 2003) and these lipid metabolites have been implicated in the pathogenesis of insulin resistance (Schooneman *et al*., 2013). In both genotypes, the muscle content of various ceramide species (C24, C24:1, and C26:1), total major ceramide (sum of C18, C18:1, C22, C24, and C24:1), and sphingolipids (Sph and dhSph) increased with aging (Fig. 2A). Elevated content of these metabolites in aged mice was associated with increased mRNA levels of the *sptlc1* gene that encodes one of the two subunits of serine palmitoyltransferase (SPT) (Fig. 2B). This enzyme catalyzes the first and rate‐limiting step in the *de novo* synthesis of sphingolipids. MISR mice of all age groups had lower C18, C18:1, and total major ceramide content, compared to WT (Fig. 2A). The muscle content of C14, C16, C20, C20:1, C20:4, C22, C22:1, and C26 ceramides did not differ based on genotype or age group (data not shown).

A subset of small‐ to medium‐chain acylcarnitines (valerylcarnitine, hexanoylcarnitine, octanoylcarnitine, cis‐4‐decenoylcarnitine, and decanoylcarnitine) was lower in MISR compared to WT mice in most age comparisons (Fig. 2C). Muscle from MISR mice had lower mRNA levels of the *aldh9a1* gene that encodes 4‐N‐trimethylaminobutyraldehyde dehydrogenase (TMABA‐DH) (Fig. 2D). This enzyme converts 4‐N‐trimethylaminobutyraldehyde into 4‐N‐trimethylaminobutyrate, a key step in carnitine synthesis. MISR mice also had corresponding reductions in carnitine muscle content (Fig. 2E) and increased levels of lysine, a precursor of carnitine biosynthesis (Fig. 2F). There were no differences in hydroxybutyrylcarnitine, linoleoylcarnitine, myristoleoylcarnitine, myristoylcarnitine, oleoylcarnitine, palmitoylcarnitine, and stearoylcarnitine muscle content between genotypes or age groups (data not shown).

Muscle content of total and major (sum of C16:0/18:0, C16:0/18:1, C16:1/18:1, C18:0/18:1, C18:0/18:2, Di‐C16:0, and Di‐C18:1) diacylglycerols (DAG) did not differ based on age or genotype (Fig. S6A). The content of some individual DAG species varied based upon age. Di‐C18:0 DAG was highest in aged (33‐month‐old) mice, C16:1/18:1 DAG, C16:1/24:1 DAG, and Di‐C18:1 DAG were highest in middle‐aged (13‐month‐old) mice, and C18:1/24:0 DAG and Di‐C16:0 DAG decreased with age (Fig. S6B). There were no statistically significant differences in the content of DAG species between genotypes.

### Sustained inactivation of NFκB is detrimental to muscle health

**Figure 3:**
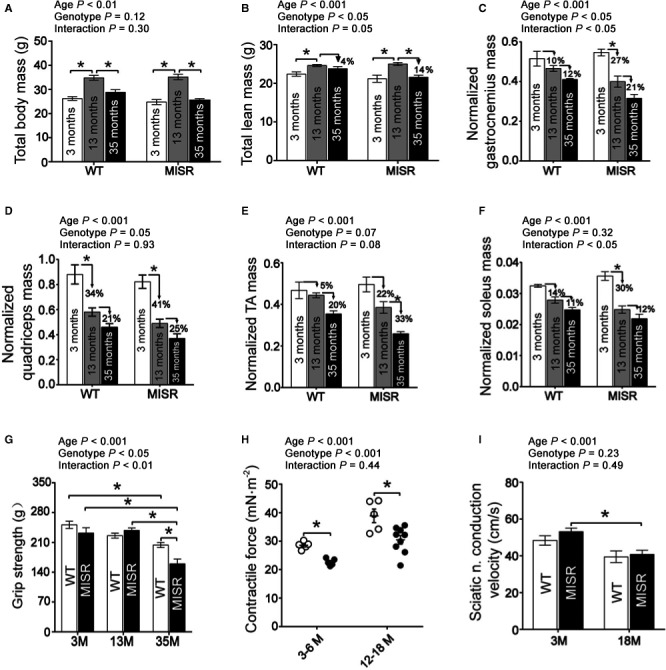
NFκB suppression leads to reduced muscle mass and function. (A) Whole‐body mass and (B) total lean mass in male wild‐type (WT) and MISR mice (cohort 1; *n *= 9–20 per group). Wet weight (normalized to total weight) of (C) gastrocnemius, (D) quadriceps, (E) tibialis anterior (TA), and (F) soleus muscles; *n *= 5 per group. (G) Grip strength; *n *= 9–20 per group. (H) Force generation in electrically stimulated hindlimb muscles in WT (white) and MISR (black) mice; *n *= 5–9 per group (I) Sciatic nerve conduction velocity; *n *= 5–6 per group. All mice were male. *P <* *0.05 by two‐way ANOVA followed by Tukey's post hoc test. All data are means ± SE.

The effect of NFκB suppression on muscle mass was first assessed in 3‐, 13‐, and 35‐month‐old male mice. Total and whole‐body lean mass, measured by quantitative magnetic resonance (QMR), increased in both genotypes from 3 to 13 months (Fig. 3A,B). As the mice aged further (35 months old), total mass and whole‐body lean mass decreased in both genotypes. Consistent with these findings, wet weights of gastrocnemius, quadriceps, tibialis anterior, and soleus muscles declined with age progression in WT and MISR mice (Fig. 3C–F). We hypothesized that NFκB inactivation would protect against aging‐induced sarcopenia. However, against our hypothesis, whole‐body lean mass was lower in MISR compared with WT mice (Fig. 3B). Moreover, the magnitude of the aging‐induced decline in whole‐body lean mass (Fig. 3B) and in the weights of some muscles (gastrocnemius and soleus) (Fig. 3C–F) was higher in MISR than WT mice. As the reduction in muscle mass with NFκB inactivation was unexpected, we aged a second cohort of male and female mice and measured lean mass by QMR. In line with the findings from the first cohort, whole‐body lean mass was lower in male MISR mice (Fig. S7A). In addition, reduced lean mass also was seen in female MISR mice (Fig. S7B).

Sarcopenia associated with aging manifests as decreases in muscle mass and neuromuscular function, but they do not necessarily change to the same degree over time. Thus, we tested the effects of NFκB inactivation on neuromuscular function by measuring grip strength, contractile force, and nerve conduction velocity in hindlimb (primarily gastrocnemius) muscles from male mice. Consistent with the muscle mass measurements, grip strength (combined forelimb and hindlimb) decreased with advancing age in both genotypes, and grip strength was 22% lower in aged (35‐month‐old) MISR compared with age‐matched WT animals (Fig. 3G). Hindlimb force generation increased in both genotypes as mice matured from 3–6 to 12–18 months of age (Fig. 3H). Muscles of 3‐ to 6‐month‐old MISR mice generated 20% less force than age‐matched WT mice and muscles of 12‐ to 18‐month‐old MISR mice generated 22% less force than age‐matched WT mice. Sciatic nerve conduction velocity was assessed in 3‐ and 18‐month‐old mice. Taking together all groups, aging leads to a significant reduction in conduction velocity (Fig. 3I). Compared with 3‐month‐old mice from the same genotype, conduction velocity was 18% lower in 18‐month‐old WT mice and 23% lower in 18‐month‐old MISR mice. Sciatic nerve conduction velocity was not significantly different between WT and MISR mice.

### NFκB inhibition alters the expression of several genes involved in muscle growth and atrophy

**Figure 4:**
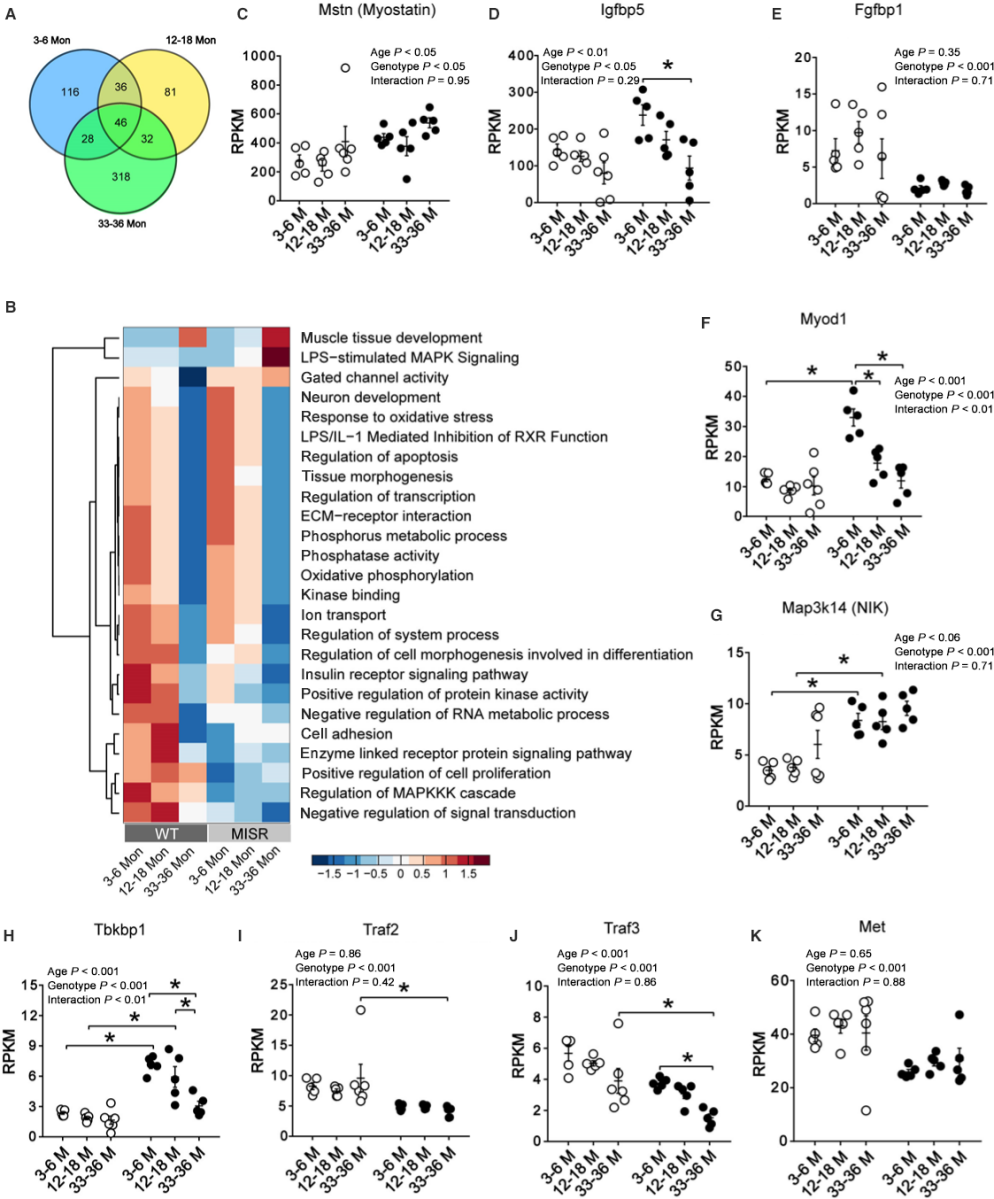
Genes and pathways regulated by aging and NFκB suppression. (A) Number of differentially expressed genes (DEGs) in WT vs. MISR (*P *<* *0.05). Gene lists in these groups are provided in Table S1 (Supporting information) . (B) Gene Ontology and IPA analyses of pathways/gene sets differentially regulated by aging and NFκB suppression (p‐values and gene lists for each corresponding functions are provided in Table S2). (C–K) mRNA levels of *mstn*,* igfbp5*,* fgfbp1, myod1*,* map3k14* (NIK), *tbkbp1*,* traf2*,* traf3*, and *c‐met* in quadriceps muscle from WT (white) and MISR (black) mice. *n *= 5–6 per group. Data analyzed by two‐way ANOVA; *P <* *0.05 by Tukey's post hoc test. All data are means ± SE.

As NFκB modulates the expression of hundreds of genes, we conducted whole‐genome RNA sequencing to facilitate the detection of alterations in gene expression that could explain reduced muscle size and strength in MISR mice. The total number of genes differentially regulated in WT vs. MISR was 659 (*P*‐value <0.05); 228 genes changed in 3‐ to 6‐, 197 in 12‐ to 18‐, and 424 in 33‐ to 36‐month‐old mice (Figs 4A, and S8A for differentially expressed genes selected with adjusted *P*‐value <0.05). Figure 4B shows the top signaling pathways involved in muscle growth/development differentially expressed during aging in both WT and MISR mice and pathways differentially expressed between genotypes. A heatmap with all pathways/functional gene sets is shown in Fig. S8B. In addition to analyzing changes in gene sets, we focused on specific genes involved in muscle growth and atrophy. NFκB suppression resulted in significantly elevated expression of *mstn* (myostatin) (Fig. 4C), a potent inhibitor of muscle growth. NFκB inhibition also led to higher expression of insulin‐like growth factor binding protein (*IGFBP*)5, which gene product suppresses muscle growth (Salih *et al*., 2004) (Fig. 4D). Finally, MISR mice had lower expression of fibroblast growth factor binding protein (*Fgfbp*)1, which plays a key role in neuromuscular junction (NMJ) formation and function (Williams *et al*., 2009) (Fig. 4E).

### Altered expression of genes that modulate muscle progenitor cell migration, differentiation, and fusion

NFκB suppression resulted in higher expression of *myod1* (MyoD) (Fig. 4F), a master regulator of myogenesis that promotes muscle cell differentiation. NFκB suppression also led to changes in expression of genes that encode for proteins within the noncanonical NFκB pathway, which promotes skeletal muscle cell differentiation and fusion (Enwere *et al*., 2012). Changes included higher mRNA levels of *map3k14* (NFκB‐inducing kinase; NIK) (Fig. 4G) and *Tbkbp1* (Fig. 4H), and lower mRNA levels of *Traf2* (Fig. 4I) and *Traf3* (Fig. 4J). Lastly, MISR mice of all age groups had lower mRNA levels of *c‐met* (Fig. 4K), a gene that encodes MET, a tyrosine kinase receptor essential for muscle progenitor cell delamination and migration.

### *In vitro* NFκB inhibition accelerates muscle cell differentiation

**Figure 5:**
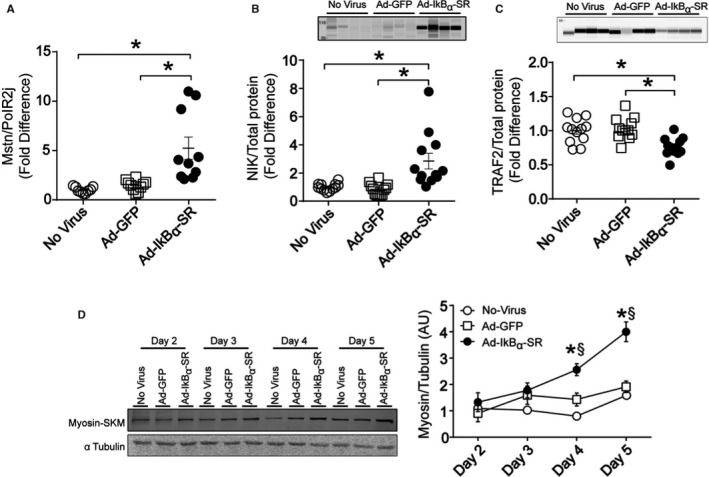
NFκB suppression promotes muscle cell differentiation. C2C12 myoblasts were transduced with Ad‐IκBα‐SR or Ad‐GFP, as described in the Methods. (A) *Mstn* mRNA level was measured by real‐time RT–PCR. NIK (B) and TRAF2 (C) protein content was measured by capillary electrophoresis. *P <* *0.05 from two‐way ANOVA followed by Tukey's post hoc test. Data are means ± SE from three independent experiments performed with an *n *= 2–6 per condition per experiment. (D) C2C12 myoblasts were transduced with Ad‐IκBα‐SR or Ad‐GFP. Fast myosin (SKM) heavy‐chain protein was measured by Western blotting during progressing differentiation stages. *P <* *0.05 Ad‐IκBα‐SR vs. Ad‐GFP and §*P *<* *0.05 Ad‐IκBα‐SR vs no‐virus from two‐way ANOVA followed by Tukey's post hoc test. Data are means ± SE from one experiment done with an *n *= 4 per condition, and the experiment was repeated two more times with similar results.

Adenoviral‐mediated overexpression of an IκBα super‐repressor (SR) mutant (S32A/S36A) in C2C12 myoblasts recapitulated the *in vivo* changes in expression of some genes and proteins involved in muscle differentiation, growth, and atrophy, including enhanced myostatin gene expression (Fig. 5A). NFκB suppression with the IκB‐SRα mutant also led to increased NIK (Fig. 5B) and lower TRAF2 (Fig. 5C) protein levels, reflecting increased flux through the noncanonical NFκB pathway. Inhibition of NFκB in C2C12 myoblasts promoted early differentiation, shown by increased expression of fast myosin (SKM) heavy chain at earlier time points (Fig. 5D). C2C12 cell proliferation was not affected by the SR mutant (Fig. S9).

### Elevated proteasome activity in MISR mice

**Figure 6:**
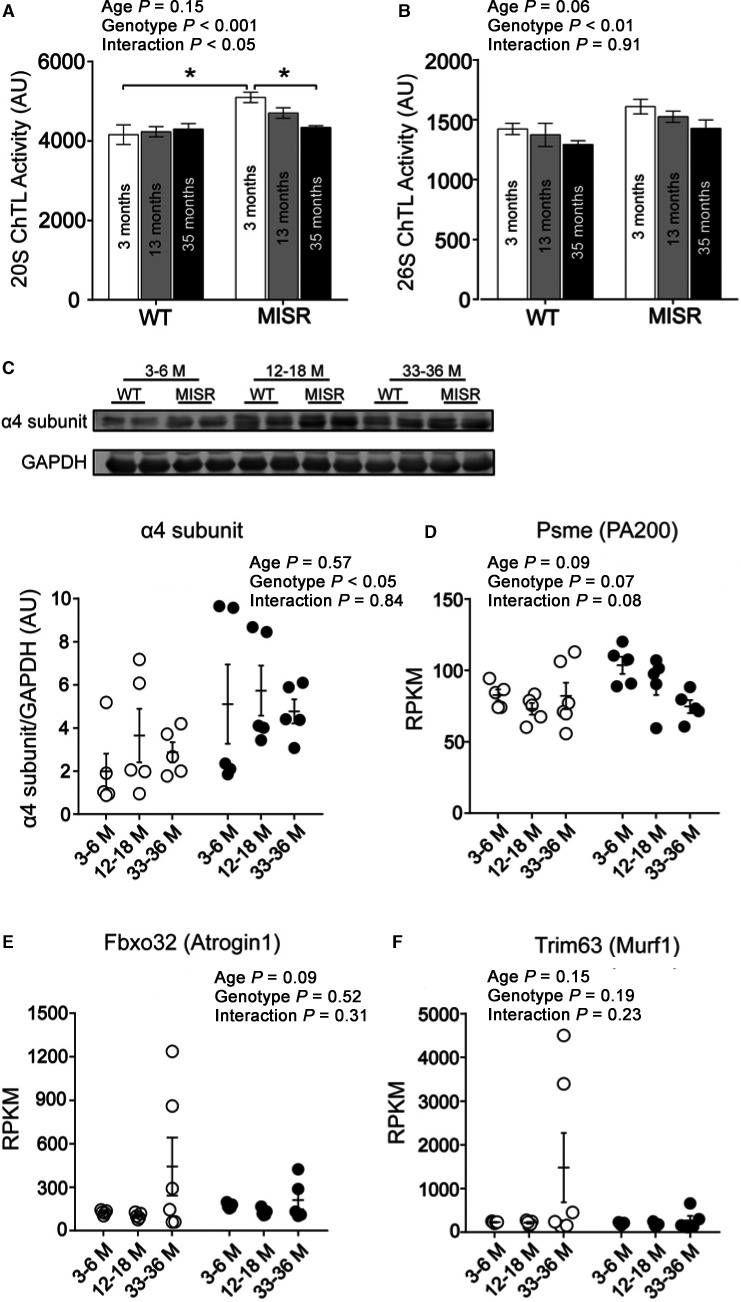
Elevated proteasome activity in MISR mice. (A) 20S and (B) 26S proteasome activity in quadriceps muscle from WT and MISR male mice. *n *= 4 per group. (C) Total proteasomal protein content represented by the α4 subunit of the 20S proteasome core in quadriceps muscle from WT (white) and MISR (black) mice. *n *= 5 per group. (D) *Psme4* (PA200), (E) *fbxo32* (atrogin), and (F) *trim63* (MuRF1) mRNA levels in quadriceps. *n *= 5–6 per group. Data analyzed by two‐way ANOVA; *P <* *0.05 by Tukey's post hoc test. All data are means ± SE.

As proteasomes are the primary sites for protein degradation in muscle, we measured activity of 26S (the most abundant proteasome) and 20S (its proteolytic core) in muscle from WT and MISR mice. The activities of 26S and 20S were increased in MISR mice, and these differences were more pronounced in young (3‐ to 6‐month‐old) and middle‐aged (12‐ to 18‐month‐old) mice (Fig. 6A,B). In parallel, the total content of proteasomal protein represented by the α4 subunit of the 20S proteasome core also was higher in muscle from MISR mice (Fig. 6C). In 3‐ to 6‐ and 12‐ to 18‐month‐old MISR mice, elevated proteasome activity was associated with a trend (*P *=* *0.07) for higher expression of *psme4* (Fig. 6D), which encodes proteasome activator (PA)200. Transcript levels of the E3 ubiquitin ligases *fbxo32* (atrogin) and *trim63* (MuRF1) were not significantly different between age and genotype groups (Fig. 6E,F). Gene expression levels of several proteasomal subunits increased with aging, but there was no difference between WT and MISR mice (Fig. S10). In addition to the proteasome, the autophagy–lysosome pathway is another major regulator of muscle protein degradation. We found that the expression of several genes in the autophagy–lysosome pathway—*Foxo3, Ctsl, Lamp2, map1lc3a* (LC3A), *map1lc3b* (LC3B), *Atg3*, and *Atg7*—were increased with aging, whereas *map1lc3a* (LC3A) expression was higher in MISR mice (Fig. S11A–G). Consistent with the increase in autophagy genes, aging (in WT and MISR) resulted in higher LC3‐II/LC3‐I ratio, which is another marker suggestive of increased autophagy (Fig. S11H).

## Discussion

Multiple lines of evidence indicate that NFκB is activated during aging, and suggest that inhibition of this pathway may be effective in slowing down aging and age‐related diseases. For example, Adler *et al*. identified NFκB as a candidate driver of aging‐related transcriptional changes in multiple human and mouse tissues (Adler *et al*., 2007) and showed that genetic blockade of NFκB protected the skin from the effects of aging (Adler *et al*., 2008). Zhang *et al*. reported that hypothalamic‐specific overexpression of the super‐repressor IκBα mutant in mice had global anti‐aging effects, including prolonging lifespan, improving cognition, and increasing dermal thickness, bone density, muscle size, exercise endurance, and tendon resistance, whereas overexpression of constitutively active IKKβ had opposite effects (Zhang *et al*., 2013). Thus, we predicted that suppression of NFκB in muscle would be protective against aging‐induced insulin resistance and sarcopenia, two common age‐related disorders. However, while NFκB inhibition improved insulin resistance in aged mice, it did not slow down aging‐related muscle loss and had a detrimental effect on muscle mass and strength.

The sarcopenic phenotype in MISR mice was unexpected because sustained stimulation of the classical IKK‐NFκB pathway through robust (10–15 fold) overexpression of IKKβ leads to severe muscle atrophy (Cai *et al*., 2004). In addition, NFκB is thought to protect muscle from various atrophy‐inducing insults. Cai *et al*. reported that muscle from MISR mice did not become atrophic when subjected to denervation and cancer (Cai *et al*., 2004). Protection from denervation, unloading, immobilization, endotoxin administration, and cryoinjury also have been reported using different models of suppressed NFκB signaling (Hunter & Kandarian, 2004; Mourkioti *et al*., 2006; Judge *et al*., 2007; Van Gammeren *et al*., 2009; Haegens *et al*., 2012; Okazaki *et al*., 2014; Oh *et al*., 2016). The resistance conferred by NFκB axis suppression to these atrophy‐inducing stimuli indicates that NFκB has opposing effects (both negative and positive) on muscle development and health, depending on the level of flux (*i.e*., dosage) through the pathway. Our findings also indicate that the mechanism behind the muscle loss seen with aging differs from muscle wasting in other pathologic conditions (cancer, denervation, disuse, *etc*.). In line with this notion, aging did not upregulate the expression of atrogin and MuRF1, ubiquitin ligases implicated in the atrophy caused by denervation and cancer (Bodine *et al*., 2001; Cai *et al*., 2004; Sacheck *et al*., 2007). While the basis for aging‐induced sarcopenia is not fully understood, our results point toward alterations in the autophagy–lysosome pathway, which is another key cellular mechanism involved in protein degradation (Wohlgemuth *et al*., 2010).

Our global transcriptomics approach to facilitate identification of potential molecular changes associated with decreased muscle mass revealed that MISR mice had altered expression of many genes that impact muscle (Summarized in Fig. S12), indicating that the atrophy is multifactorial. MISR mice had elevated levels of myostatin, a member of the TGFβ protein family that is a potent inhibitor of muscle growth (McPherron *et al*., 1997). MISR mice also had elevations in *igfbp5* and reductions in *c‐met* gene expression. IGFBP5 is a ubiquitously expressed IGF binding protein with high affinity for IGF1, a growth factor essential for muscle mass attrition. Transgenic mice overexpressing IGFBP5 suffer from growth retardation and muscle atrophy, due to suppressed IGF1 activity (Salih *et al*., 2004). MET (encoded by *c‐met*) is a tyrosine kinase receptor that interacts with its ligand hepatocyte growth factor (also known as scatter factor) and produced by nonsomitic mesodermal cells to delineate the migratory route of muscle progenitor cells. The importance of MET in muscle development is underscored by findings that mouse embryos lacking functional MET have underdeveloped skeletal muscle tissue in the limbs (Bladt *et al*., 1995). MISR mice of all ages also had a significant reduction in the mRNA level of *fgfbp1*, which plays a critical role in cell differentiation and migration by binding to fibroblast growth factors and potentiating their biological effect (Williams *et al*., 2009). FGFBP1 also promotes normal development and function of the NMJ (Williams *et al*., 2009).

MISR mice had enhanced expression of MyoD, a myogenic factor that is a key driver of muscle differentiation. The increase in MyoD is in line with work from Guttridge *et al*. that have reported that NFκB downregulates MyoD expression (Guttridge *et al*., 2000). MISR mice also displayed changes in genes that encode for proteins within the noncanonical NFκB pathway, including increases in *map3k14* (NIK) and *Tbkbp1*, and decreases in TRAF2 and TRAF3. NIK is a central component of the noncanonical NFκB pathway that integrates signals from TNF receptor family members. NIK is subjected to a fate control mechanism, such that the basal level of NIK is kept low by the TRAF proteins. Therefore, a decrease in TRAF2 and TRAF3, as seen in MISR mice, would be consistent with increased flux via this pathway. The noncanonical NFκB pathway promotes fusion (Enwere *et al*., 2012; Hindi *et al*., 2013), is elevated in muscle cells from older human subjects (Urban *et al*., 2014), and has been associated with muscle wasting (Li *et al*., 2009). Moreover, the findings from the cell culture experiments indicate that NFκB suppression accelerates myoblast differentiation *in vitro*. Thus, it is possible that increased expression of MyoD and enhanced flux via the noncanonical NFκB pathway may lead to premature differentiation and fusion *in vivo*. While gene expression levels of myosin heavy chains were not different between genotypes (Fig. S13), a more detailed analysis of muscle cell differentiation across the lifespan in MISR mice *in vivo* will be required to firmly establish whether premature fusion contributed to the decrease in muscle mass. Lastly, NFκB is thought to control satellite cell function and their dysfunction during development could have resulted in reduced muscle mass. Yet, this possibility seems less likely because myogenic colony formation efficiency and satellite cell frequency are not affected in young MISR mice (Oh *et al*., 2016).

In addition to the altered expression of several genes involved in muscle development, growth, and atrophy, muscle from MISR mice had elevated proteasome activity. One mechanism behind the increase in activity is the elevation in total content of protein of the proteasome catalytic core, present in all enzymatically active proteasome assemblies. Another potential mechanism behind the increase in proteasome activity in MISR mice is enhanced expression of *psme4* (PA200), which binds to proteasomes to stimulate the hydrolysis of peptides (Ustrell *et al*., 2002). It is also possible that NFκB suppression leads to changes in the abundance of distinct forms of the proteasomes, each with specific activities and functions, from the 26S super‐assemblies to the free 20S cores.

The insulin clamping experiments uncovered insulin resistance in aged WT mice that was not readily apparent upon glucose tolerance testing. Increased intramyocellular ceramide content likely contributed to reduced insulin action in aged mice, as ceramides directly inhibit Akt. The elevation in muscle ceramide content was accompanied by an age‐mediated increase in the level of one of the two subunits of SPT, the rate‐limiting enzyme that controls *de novo* synthesis of sphingolipids. Moreover, NFκB suppression led to decreases in ceramide and acylcarnitine content, which likely contributed to the improvements in insulin action. Aged MISR mice also had lower level of the acylcarnitine precursor carnitine, likely as a result of lower expression of *aldh9a1* (TMABA‐DH), a key carnitine biosynthetic enzyme. As variations in muscle fiber types also can affect glucose metabolism and insulin action, we compared fiber‐type distribution between groups. WT and MISR mice did not have significant differences in fiber‐type distribution, whereas aging led to a small increase in the proportion of IIa/x vs. IIb fibers (Fig. S14).

Another unexpected finding in this study was the dissociation between muscle mass and insulin action in MISR mice. That is*,* MISR mice had improved insulin sensitivity despite having lower muscle mass. Insulin action/sensitivity modulates muscle mass through mTOR‐regulated protein synthesis. Conversely, sarcopenia is thought to promote insulin resistance (reduced insulin‐mediated whole‐body glucose disposal) because muscle tissue is the main site responsible for glucose disposal, and sarcopenia promotes physical inactivity. Improved insulin sensitivity in MISR mice may have been a compensatory mechanism in response to the negative effects of NFκB suppression on muscle mass. It is also possible that insulin‐mediated glucose disposal and muscle mass control are not inextricably linked.

NFκB suppression resulted in altered expression of multiple genes and this finding was anticipated because this transcription factor is at the nexus of numerous signaling pathways. Changes in gene expression in MISR mice are likely caused by direct and indirect mechanisms. For example, the 5′‐regulatory region of myostatin has the consensus sequence of the NFκB binding site (Ma *et al*., 2001). Thus, based upon our results, NFκB appears to function as a transcriptional repressor of the myostatin gene. NFκB also has been reported to suppress *fgfbp1* promoter activity (Rosli *et al*., 2013). In addition to these documented reports of transcriptional regulation by NFκB, the promoter regions of several genes altered in MISR mice have predicted NFκB binding motifs, including *slc22a5*,* igfbp5*,* map3k14* (NIK), *tbkbp1*,* traf2*, and *foxo3*. Besides direct transcriptional regulation by NFκB, gene expression changes in MISR mice also could be mediated via indirect mechanisms. For example, MISR mice had increased expression levels of the transcription factor *myod1* and the promoter regions of some genes altered in MISR mice have predicted *myod1* binding sites, including *slc22a5*,* traf2*,* map1lc3a*, and *map1lc3b*. The *c‐met* promoter region is regulated by peroxisome proliferator‐activated receptor (PPAR)γ (Kitamura *et al*., 1999), a transcription factor/nuclear receptor that can interact directly with NFκB (Chung *et al*., 2000). The *aldh9a1* (TMABA‐DH) (Wen *et al*., 2012) promoter also is modulated by PPARα, which cross talks with NFκB (Delerive *et al*., 1999). Various other genes altered in MISR mice have predicted PPARγ (*slc22a5*,* myod1, igfbp5, map3k14*,* tbkbp1*,* traf2, foxo3, lamp2,* and *map1lc3a and map1lc3b*) and PPARα (*slc22a5*,* myod1, igfbp5, map3k14*, and *tbkbp1*) binding motifs.

In conclusion, sustained NFκB suppression confers some beneficial effects on glucose homeostasis (*i.e*., insulin sensitivity), likely due to reduced levels of ceramides and acylcarnitines in muscle. However, our findings indicate that NFκB plays an important role in muscle development and maintenance throughout the lifespan. Moreover, we demonstrate that sustained (lifelong) NFκB suppression has harmful effects on muscle mediated by increased proteasome activity and altered expression of several genes involved in muscle growth and atrophy. Future research could test whether a more subtle intervention to block this pathway (*e.g*., pharmacological), applied at a different time in life, or for less time (*e.g*., inducible genetic approach), can improve muscle function without impairing its growth.

## Experimental procedures

### MISR mice

Generation of these mice and genotyping procedures were described previously (Cai *et al*., 2004). All procedures were approved by the Institutional Animal Care and Use Committee at the University of Texas Health Science Center at San Antonio (UTHSCSA).

### Intraperitoneal glucose tolerance test

Mice were injected intraperitoneally with dextrose (1.5 g kg^−1^). Glucose concentrations were measured in tail blood at times 0, 30, 60, 90, and 120 min using an automatic glucose meter (Roche Diagnostics, Indianapolis, IN, USA). Food was removed 8–10 h before the experiments.

### Hyperinsulinemic, euglycemic clamp studies

Under anesthesia with inhaled isoflurane, a catheter was inserted into the right atrium of the heart through the jugular vein. Three to five days later, a 90‐min euglycemic hyperinsulinemic (18 mU kg^−1^ min^−1^) clamp with tritiated glucose was performed in fasting mice, as described previously (Liang *et al*., 2009). At the end of the insulin clamp, quadriceps muscles were removed and snap‐frozen for insulin signaling assays. Plasma insulin concentrations were measured using an ELISA kit (Crystal Chem Inc, Downers Grove, IL, USA). The M value was calculated as the GIR minus the glucose space.

### QMR

Lean mass was determined by QMR (EchoMRI‐100, Houston, TX, USA) in awake mice.

### Muscle mass measurement

For muscle tissue harvesting, mice were anesthetized (80 mg kg^−1^ ketamine and 10 mg kg^−1^ xylazine, i.p.) and euthanized by cervical dislocation. Individual muscles were carefully dissected and weighed to 0.1 mg accuracy.

### Grip strength

The combined strength of forelimbs and hindlimbs was measured using a Grip Strength Meter with mesh grid pull bar (Columbus Instruments 1027 CSM, Columbus, OH) specifically designed for mice. Mice were allowed to grasp the pull bar with forelimbs and hindlimbs and were then gradually pulled backward in a horizontal plane until they lost their grip. Maximum grip strength is recorded on the device. Mice are not trained prior to testing. The highest value from five consecutive trials is designated the mouse's maximum grip strength.

### Contractile force

Maximal isometric torque of the posterior crural muscles (gastrocnemius, soleus, plantaris) was measured *in vivo* in 3‐ to 6‐ and 12‐ to 18‐month‐old male mice. Mice were anesthetized using inhaled isoflurane. Mice rested in a prone position on a heated (37 °C) platform. The knee was fixed with clamps with the foot attached to a footplate located on the shaft of a servomotor that measures changes in force (model 1300A; Aurora Scientific, Aurora, ON, Canada). Two stainless steel subdermal electrodes were inserted subcutaneously in the body of the posterior crural muscles. Peak isometric torque was measured in response to voltage delivered by an electrical stimulator (model 701C; Aurora Scientific) at increasing frequencies (20, 40, 60, 80, 100, and 150 Hz).

### Proteasome activity

Muscle tissues were homogenized in ice‐cold proteasome homogenization buffer (50 mm Tris‐HCl, pH7.5, 0.05% NP‐40, 0.25 mm sucrose, 1 mm DTT, 2 mm ATP, 5 mm MgCl_2_), followed by centrifugation at 10,000 *g* for 20 min at 4 °C. Supernatant was transferred to a new tube, and protein concentrations were determined; 40 μg of proteins per sample per assay was used in all assays, which were performed in triplicates and repeated at least once. Chymotrypsin‐like proteasome activity was measured in reaction buffer which contained 40 μm of substrate Suc‐LLVY‐AMC (EMD, Gibbstown, NJ, USA) in 50 mm Tris‐HCL, pH 7.5, 0.25 mm sucrose, 1 mm DTT, and 5 mm MgCl_2_. The proteasome inhibitor MG‐132 at 1 μm (Sigma‐Aldrich, St. Louis, MO) was included in a separate reaction to monitor nonspecific background reactions. For 20S chymotrypsin‐like activity assay, a final concentration of 0.03% SDS was included in the reaction buffer. For 26S activity assay, 46 μm ATP was added into the reaction mixture without SDS. The reaction was monitored for 120 min at 37 °C by measuring the fluorescence intensity in a 96‐well plate reader at the following wave lengths: excitation = 380 nm, emission = 440 nm. Proteasome activity at the 60‐min time point was calculated by subtracting nonspecific background values obtained in the presence of inhibitors from those without inhibitors.

### C2C12 cell culture experiments

Cell culture experiments were carried out in 6‐well plates with growth media (DMEM with 10% FBS and 1% penicillin/streptomycin) seeded with 2 × 10^4^ cells well^−1^. C2C12 myoblasts at 70% confluence were transduced for 12 h with 10^8^ IU well^−1^ Ad‐IκBα‐SR or Ad‐GFP (Vector Biolabs, Philadelphia, PA, USA). We have confirmed the ability of this adenoviral vector to inhibit NFκB signaling in cultured muscle cells (Reyna *et al*., 2008). To induce differentiation, the media was then changed to DMEM with 2% FBS. For measurement of fast myosin skeletal heavy chain over time, cells were differentiated with 2% HS. Cells were harvested 3 days later with lysis buffer (20 mm Tris, pH 7.5, 5 mm EDTA, 10 mm Na_3_PO_4_, 100 mm NaF, 2 mm Na_3_VO_4_, 1% Nonidet P‐40, 10 μm leupeptin, 3 mm benzamidine, 10 μg mL^−1^ aprotinin, and 1 mM phenylmethylsulfonyl fluoride) or TRIzol for protein content and mRNA expression measurements, respectively. Myoblast proliferative capacity was assessed using a CellTiter 96 AQueous One Solution Proliferation Assay (Promega, Sunnyvale, CA, USA). Cells were seeded at 2000 cells well^−1^ and transduced in 2% FBS differentiation media with Ad‐10^7 ^IU well^−1^ IκBα‐SR or Ad‐GFP for 12 h before being switched to growth media for measurements of cell proliferation over time.

### Statistical analysis

All data are presented as the mean ± SE. Comparisons of means between groups were done by two‐way ANOVA followed by the Tukey's test or unpaired two‐tailed t‐test, as specified in the figure legends. GraphPad Prism 7 (GraphPad Software, La Jolla, CA) was used for statistical analyses.

### Other methods

The Supplemental Information section provides detailed descriptions of the indirect calorimetry and spontaneous activity measurements, nerve conduction studies, metabolomics, real‐time PCR, Western blotting, fiber typing, capillary electrophoresis, RNA sequencing procedures, and gene expression data analyses.

## Funding

This work was supported by grants from the NIH (R01‐DK80157 and R01‐DK089229), and the American Diabetes Association to N.M. A.B. was supported by an award from the NIH (K01AG038555) and an award from the American Federation for Aging Research. J.V. and M.E.W. were supported by a Biology of Aging T32 Training Grant (T32 AG021890), and Y.C. was supported by NCI Cancer Center Shared Resources NCI P30CA54174 and NIH CTSA 1UL1RR025767‐01. This research also was supported by the San Antonio Nathan Shock Center of Excellence on Aging Biology (P30 AG013319), by the Lipidomics Shared Resource, Hollings Cancer Center, Medical University of South Carolina (MUSC) (P30 CA138313) and the Lipidomics Core in the South Carolina Lipidomics and Pathobiology COBRE, Department of Biochemistry, MUSC (P20 RR017677).

## Conflict of interest

None declared.

## Author contributions

N.Z., J.V., Y.Z, M.E.L., Y.Z., A.B., M.E.W., and H.Y. designed and conducted experiments, analyzed and interpreted data, prepared figures, and edited the manuscript. K.E.F, S.N.A, P.O., M.G., H.V.R., and S.E.S. designed experiments, interpreted data, and edited the manuscript. Y.C. and H.H.C. analyzed and interpreted data, prepared figures, and edited the manuscript. N.M. directed the project, designed experiments, analyzed and interpreted data, prepared figures, and wrote the manuscript.

## Supporting information


**Fig. S1** mRNA transcript level of *nfkb1a* in quadriceps muscle from WT (white) and MISR (black) mice.
**Fig. S2** Glucose tolerance testing.
**Fig. S3** Blood glucose (A) and plasma insulin (B) concentrations during the clamp.
**Fig. S4** Akt phosphorylation (Ser473) in the baseline (non‐insulin stimulated) state assessed by Western blotting in quadriceps muscle.
**Fig. S5** Indirect calorimetry and spontaneous activity measurement.
**Fig. S6** Diacylglycerol (DAG) content in quadriceps muscle.
**Fig. S7** Lean mass in male and female mice (Cohort 2).
**Fig. S8** Differentially expressed genes selected at higher stringent criterion and the extended gene sets and biology pathways regulated by aging and NFκB suppression.
**Fig. S9** NFκB suppression does not affect myoblast proliferative capacity.
**Fig. S10** Proteasomal protein gene expression significantly changed with age.
**Fig. S11** Aging upregulates the autophagy‐lysosome pathway.
**Fig. S12** Mechanism underlying reduced muscle mass in MISR mice.
**Fig. S13** Gene expression levels of myosin heavy chains in quadriceps muscle.
**Fig. S14** Myosin heavy chain profile.

**Table S1** (a) Differentally expressed genes (DEGs) comparing MISR/WT at 3–6 months of age. (b) Differentally expressed genes (DEGs) comparing MISR/WT at 12–18 months of age. (c) Differentally expressed gene (DEGs) comparing MISR/WT at 33–36 month of age.

**Table S2** Gene lists corresponding to Gene Ontology and IPA pathways/gene sets differentially regulated by aging and NFkB suppression (from Figure 4B).

**Data S1** Procedures and Materials.

## References

[acel12613-bib-0001] Adler AS , Sinha S , Kawahara TL , Zhang JY , Segal E , Chang HY (2007) Motif module map reveals enforcement of aging by continual NF‐kappaB activity. Genes Dev. 21, 3244–3257. 18055696 10.1101/gad.1588507 PMC2113026

[acel12613-bib-0002] Adler AS , Kawahara TL , Segal E , Chang HY (2008) Reversal of aging by NFkappaB blockade. Cell Cycle 7, 556–559. 18256548 10.4161/cc.7.5.5490

[acel12613-bib-0003] Bladt F , Riethmacher D , Isenmann S , Aguzzi A , Birchmeier C (1995) Essential role for the c‐met receptor in the migration of myogenic precursor cells into the limb bud. Nature 376, 768–771. 7651534 10.1038/376768a0

[acel12613-bib-0004] Bodine SC , Latres E , Baumhueter S , Lai VK , Nunez L , Clarke BA , Poueymirou WT , Panaro FJ , Na E , Dharmarajan K , Pan ZQ , Valenzuela DM , DeChiara TM , Stitt TN , Yancopoulos GD , Glass DJ (2001) Identification of ubiquitin ligases required for skeletal muscle atrophy. Science 294, 1704–1708. 11679633 10.1126/science.1065874

[acel12613-bib-0005] Buford TW , Cooke MB , Manini TM , Leeuwenburgh C , Willoughby DS (2010) Effects of age and sedentary lifestyle on skeletal muscle NF‐kappaB signaling in men. J. Gerontol. A Biol. Sci. Med. Sci. 65, 532–537. 20045871 10.1093/gerona/glp196 PMC2904591

[acel12613-bib-0006] Cai D , Frantz JD , Tawa NE Jr , Melendez PA , Oh BC , Lidov HG , Hasselgren PO , Frontera WR , Lee J , Glass DJ , Shoelson SE (2004) IKKbeta/NF‐kappaB activation causes severe muscle wasting in mice. Cell 119, 285–298. 15479644 10.1016/j.cell.2004.09.027

[acel12613-bib-0007] Chung SW , Kang BY , Kim SH , Pak YK , Cho D , Trinchieri G , Kim TS (2000) Oxidized low density lipoprotein inhibits interleukin‐12 production in lipopolysaccharide‐activated mouse macrophages via direct interactions between peroxisome proliferator‐activated receptor‐gamma and nuclear factor‐kappa B. J. Biol. Chem. 275, 32681–32687. 10934192 10.1074/jbc.M002577200

[acel12613-bib-0008] Delerive P , De Bosscher K , Besnard S , Vanden Berghe W , Peters JM , Gonzalez FJ , Fruchart JC , Tedgui A , Haegeman G , Staels B (1999) Peroxisome proliferator‐activated receptor alpha negatively regulates the vascular inflammatory gene response by negative cross‐talk with transcription factors NF‐kappaB and AP‐1. J. Biol. Chem. 274, 32048–32054. 10542237 10.1074/jbc.274.45.32048

[acel12613-bib-0009] Enwere EK , Holbrook J , Lejmi‐Mrad R , Vineham J , Timusk K , Sivaraj B , Isaac M , Uehling D , Al‐awar R , LaCasse E , Korneluk RG (2012) TWEAK and cIAP1 regulate myoblast fusion through the noncanonical NF‐kappaB signaling pathway. Sci. Signal. 5, ra75. 23074266 10.1126/scisignal.2003086

[acel12613-bib-0010] Ghosh S , Lertwattanarak R , Garduno Jde J , Galeana JJ , Li J , Zamarripa F , Lancaster JL , Mohan S , Hussey S , Musi N (2015) Elevated muscle TLR4 expression and metabolic endotoxemia in human aging. J. Gerontol. A Biol. Sci. Med. Sci. 70, 232–246. 24846769 10.1093/gerona/glu067 PMC4311182

[acel12613-bib-0011] Guttridge DC , Mayo MW , Madrid LV , Wang CY , Baldwin AS Jr (2000) NF‐kappaB‐induced loss of MyoD messenger RNA: possible role in muscle decay and cachexia. Science 289, 2363–2366. 11009425 10.1126/science.289.5488.2363

[acel12613-bib-0012] Haegens A , Schols AM , Gorissen SH , van Essen AL , Snepvangers F , Gray DA , Shoelson SE , Langen RC (2012) NF‐kappaB activation and polyubiquitin conjugation are required for pulmonary inflammation‐induced diaphragm atrophy. Am. J. Physiol. Lung Cell. Mol. Physiol. 302, L103–L110. 22003096 10.1152/ajplung.00084.2011

[acel12613-bib-0013] Helenius M , Hanninen M , Lehtinen SK , Salminen A (1996) Aging‐induced up‐regulation of nuclear binding activities of oxidative stress responsive NF‐kB transcription factor in mouse cardiac muscle. J. Mol. Cell. Cardiol. 28, 487–498. 9011632 10.1006/jmcc.1996.0045

[acel12613-bib-0014] Hindi SM , Tajrishi MM , Kumar A (2013) Signaling mechanisms in mammalian myoblast fusion. Sci. Signal. 6, re2. 23612709 10.1126/scisignal.2003832 PMC3724417

[acel12613-bib-0015] Hunter RB , Kandarian SC (2004) Disruption of either the Nfkb1 or the Bcl3 gene inhibits skeletal muscle atrophy. J. Clin. Investig. 114, 1504–1511. 15546001 10.1172/JCI21696 PMC525738

[acel12613-bib-0016] Judge AR , Koncarevic A , Hunter RB , Liou HC , Jackman RW , Kandarian SC (2007) Role for IkappaBalpha, but not c‐Rel, in skeletal muscle atrophy. Am. J. Physiol. Cell Physiol. 292, C372–C382. 16928772 10.1152/ajpcell.00293.2006

[acel12613-bib-0017] Kitamura S , Miyazaki Y , Hiraoka S , Toyota M , Nagasawa Y , Kondo S , Kiyohara T , Shinomura Y , Matsuzawa Y (1999) PPARgamma inhibits the expression of c‐MET in human gastric cancer cells through the suppression of Ets. Biochem. Biophys. Res. Commun. 265, 453–456. 10558888 10.1006/bbrc.1999.1715

[acel12613-bib-0018] Korhonen P , Helenius M , Salminen A (1997) Age‐related changes in the regulation of transcription factor NF‐kappa B in rat brain. Neurosci. Lett. 225, 61–64. 9143018 10.1016/s0304-3940(97)00190-0

[acel12613-bib-0019] Li H , Mittal A , Paul PK , Kumar M , Srivastava DS , Tyagi SC , Kumar A (2009) Tumor necrosis factor‐related weak inducer of apoptosis augments matrix metalloproteinase 9 (MMP‐9) production in skeletal muscle through the activation of nuclear factor‐kappaB‐inducing kinase and p38 mitogen‐activated protein kinase: a potential role of MMP‐9 in myopathy. J. Biol. Chem. 284, 4439–4450. 19074147 10.1074/jbc.M805546200 PMC2640955

[acel12613-bib-0020] Liang H , Balas B , Tantiwong P , Dube J , Goodpaster BH , O'Doherty RM , DeFronzo RA , Richardson A , Musi N , Ward WF (2009) Whole body overexpression of PGC‐1alpha has opposite effects on hepatic and muscle insulin sensitivity. Am. J. Physiol. Endocrinol. Metab. 296, E945–E954. 19208857 10.1152/ajpendo.90292.2008 PMC2670619

[acel12613-bib-0021] Ma K , Mallidis C , Artaza J , Taylor W , Gonzalez‐Cadavid N , Bhasin S (2001) Characterization of 5’‐regulatory region of human myostatin gene: regulation by dexamethasone in vitro. Am. J. Physiol. Endocrinol. Metab. 281, E1128–E1136. 11701425 10.1152/ajpendo.2001.281.6.E1128

[acel12613-bib-0022] McPherron AC , Lawler AM , Lee SJ (1997) Regulation of skeletal muscle mass in mice by a new TGF‐beta superfamily member. Nature 387, 83–90. 9139826 10.1038/387083a0

[acel12613-bib-0023] Mourkioti F , Kratsios P , Luedde T , Song YH , Delafontaine P , Adami R , Parente V , Bottinelli R , Pasparakis M , Rosenthal N (2006) Targeted ablation of IKK2 improves skeletal muscle strength, maintains mass, and promotes regeneration. J. Clin. Investig. 116, 2945–2954. 17080195 10.1172/JCI28721 PMC1626136

[acel12613-bib-0024] Oh J , Sinha I , Tan KY , Rosner B , Dreyfuss JM , Gjata O , Tran P , Shoelson SE , Wagers AJ (2016) Age‐associated NF‐kappaB signaling in myofibers alters the satellite cell niche and re‐strains muscle stem cell function. Aging 8, 2871–2896. 27852976 10.18632/aging.101098 PMC5191876

[acel12613-bib-0025] Okazaki T , Liang F , Li T , Lemaire C , Danialou G , Shoelson SE , Petrof BJ (2014) Muscle‐specific inhibition of the classical nuclear factor‐kappaB pathway is protective against diaphragmatic weakness in murine endotoxemia. Crit. Care Med. 42, e501–e509. 24933061 10.1097/CCM.0000000000000407

[acel12613-bib-0026] Petersen KF , Befroy D , Dufour S , Dziura J , Ariyan C , Rothman DL , DiPietro L , Cline GW , Shulman GI (2003) Mitochondrial dysfunction in the elderly: possible role in insulin resistance. Science 300, 1140–1142. 12750520 10.1126/science.1082889 PMC3004429

[acel12613-bib-0027] Phillips T , Leeuwenburgh C (2005) Muscle fiber specific apoptosis and TNF‐alpha signaling in sarcopenia are attenuated by life‐long calorie restriction. FASEB J. 19, 668–670. 15665035 10.1096/fj.04-2870fje

[acel12613-bib-0028] Reed SA , Senf SM , Cornwell EW , Kandarian SC , Judge AR (2011) Inhibition of IkappaB kinase alpha (IKKalpha) or IKKbeta (IKKbeta) plus forkhead box O (Foxo) abolishes skeletal muscle atrophy. Biochem. Biophys. Res. Commun. 405, 491–496. 21256828 10.1016/j.bbrc.2011.01.059 PMC3056397

[acel12613-bib-0029] Reyna SM , Ghosh S , Tantiwong P , Meka CS , Eagan P , Jenkinson CP , Cersosimo E , Defronzo RA , Coletta DK , Sriwijitkamol A , Musi N (2008) Elevated toll‐like receptor 4 expression and signaling in muscle from insulin‐resistant subjects. Diabetes 57, 2595–2602. 18633101 10.2337/db08-0038 PMC2551667

[acel12613-bib-0030] Rosli SN , Shintani T , Hayashido Y , Toratani S , Usui E , Okamoto T (2013) 1alpha,25OH2D3 down‐regulates HBp17/FGFBP‐1 expression via NF‐kappaB pathway. J. Steroid Biochem. Mol. Biol. 136, 98–101. 23104116 10.1016/j.jsbmb.2012.10.011

[acel12613-bib-0031] Sacheck JM , Hyatt JP , Raffaello A , Jagoe RT , Roy RR , Edgerton VR , Lecker SH , Goldberg AL (2007) Rapid disuse and denervation atrophy involve transcriptional changes similar to those of muscle wasting during systemic diseases. FASEB J. 21, 140–155. 17116744 10.1096/fj.06-6604com

[acel12613-bib-0032] Salih DA , Tripathi G , Holding C , Szestak TA , Gonzalez MI , Carter EJ , Cobb LJ , Eisemann JE , Pell JM (2004) Insulin‐like growth factor‐binding protein 5 (Igfbp5) compromises survival, growth, muscle development, and fertility in mice. Proc. Natl Acad. Sci. USA 101, 4314–4319. 15010534 10.1073/pnas.0400230101 PMC384738

[acel12613-bib-0033] Schooneman MG , Vaz FM , Houten SM , Soeters MR (2013) Acylcarnitines: reflecting or inflicting insulin resistance? Diabetes 62, 1–8. 23258903 10.2337/db12-0466 PMC3526046

[acel12613-bib-0034] Thalacker‐Mercer AE , Dell'Italia LJ , Cui X , Cross JM , Bamman MM (2010) Differential genomic responses in old vs. young humans despite similar levels of modest muscle damage after resistance loading. Physiol. Genomics 40, 141–149. 19903761 10.1152/physiolgenomics.00151.2009 PMC2825766

[acel12613-bib-0035] Urban RJ , Dillon EL , Choudhary S , Zhao Y , Horstman AM , Tilton RG , Sheffield‐Moore M (2014) Translational studies in older men using testosterone to treat sarcopenia. Trans. Am. Clin. Climatol. Assoc. 125, 27–42; discussion 42‐24. 25125716 PMC4112698

[acel12613-bib-0036] Ustrell V , Hoffman L , Pratt G , Rechsteiner M (2002) PA200, a nuclear proteasome activator involved in DNA repair. EMBO J. 21, 3516–3525. 12093752 10.1093/emboj/cdf333 PMC126083

[acel12613-bib-0037] Van Gammeren D , Damrauer JS , Jackman RW , Kandarian SC (2009) The IkappaB kinases IKKalpha and IKKbeta are necessary and sufficient for skeletal muscle atrophy. FASEB J. 23, 362–370. 18827022 10.1096/fj.08-114249 PMC2630783

[acel12613-bib-0038] Walter R , Sierra F (1998) Changes in hepatic DNA binding proteins as a function of age in rats. J. Gerontol. A Biol. Sci. Med. Sci. 53, B102–B110. 9520905 10.1093/gerona/53a.2.b102

[acel12613-bib-0039] Wen G , Ringseis R , Rauer C , Eder K (2012) The mouse gene encoding the carnitine biosynthetic enzyme 4‐N‐trimethylaminobutyraldehyde dehydrogenase is regulated by peroxisome proliferator‐activated receptor alpha. Biochem. Biophys. Acta. 1819, 357–365. 22285688 10.1016/j.bbagrm.2012.01.004

[acel12613-bib-0040] Williams AH , Valdez G , Moresi V , Qi X , McAnally J , Elliott JL , Bassel‐Duby R , Sanes JR , Olson EN (2009) MicroRNA‐206 delays ALS progression and promotes regeneration of neuromuscular synapses in mice. Science 326, 1549–1554. 20007902 10.1126/science.1181046 PMC2796560

[acel12613-bib-0041] Wohlgemuth SE , Seo AY , Marzetti E , Lees HA , Leeuwenburgh C (2010) Skeletal muscle autophagy and apoptosis during aging: effects of calorie restriction and life‐long exercise. Exp. Gerontol. 45, 138–148. 19903516 10.1016/j.exger.2009.11.002 PMC2829942

[acel12613-bib-0042] Xiao ZQ , Majumdar AP (2000) Induction of transcriptional activity of AP‐1 and NF‐kappaB in the gastric mucosa during aging. Am. J. Physiol. Gastrointest. Liver Physiol. 278, G855–G865. 10859214 10.1152/ajpgi.2000.278.6.G855

[acel12613-bib-0043] Yuan M , Konstantopoulos N , Lee J , Hansen L , Li ZW , Karin M , Shoelson SE (2001) Reversal of obesity‐ and diet‐induced insulin resistance with salicylates or targeted disruption of Ikkbeta. Science 293, 1673–1677. 11533494 10.1126/science.1061620

[acel12613-bib-0044] Zhang G , Li J , Purkayastha S , Tang Y , Zhang H , Yin Y , Li B , Liu G , Cai D (2013) Hypothalamic programming of systemic ageing involving IKK‐beta, NF‐kappaB and GnRH. Nature 497, 211–216. 23636330 10.1038/nature12143 PMC3756938

