# Supplementary material for: Sustained NFκB inhibition improves insulin sensitivity but is detrimental to muscle health
Source: Aging Cell. 2017 May 29;16(4):847–58. doi: 10.1111/acel.12613 (PMC5506420; doi:10.1111/acel.12613)
Supplement: Supplementary file 1 — Fig. S1 mRNA transcript level of nfkb1a in quadriceps muscle from WT (white) and MISR (black) mice. Fig. S2 Glucose tolerance testing. Fig. S3 Blood glucose (A) and plasma insulin (B) concentrations during the clamp. Fig. S4 Akt phosphorylation (Ser473) in the baseline (non‐insulin stimulated) state assessed by Western blotting in quadriceps muscle. Fig. S5 Indirect calorimetry and spontaneous activity measurement. Fig. S6 Diacylglycerol (DAG) content in quadriceps muscle. Fig. S7 Lean mass in male and female mice (Cohort 2). Fig. S8 Differentially expressed genes selected at higher stringent criterion and the extended gene sets and biology pathways regulated by aging and NFκB suppression. Fig. S9 NFκB suppression does not affect myoblast proliferative capacity. Fig. S10 Proteasomal protein gene expression significantly changed with age. Fig. S11 Aging upregulates the autophagy‐lysosome pathway. Fig. S12 Mechanism underlying reduced muscle mass in MISR mice. Fig. S13 Gene expression levels of myosin heavy chains in quadriceps muscle. Fig. S14 Myosin heavy chain profile. [file ACEL-16-847-s001.docx]

**Supplemental Data**


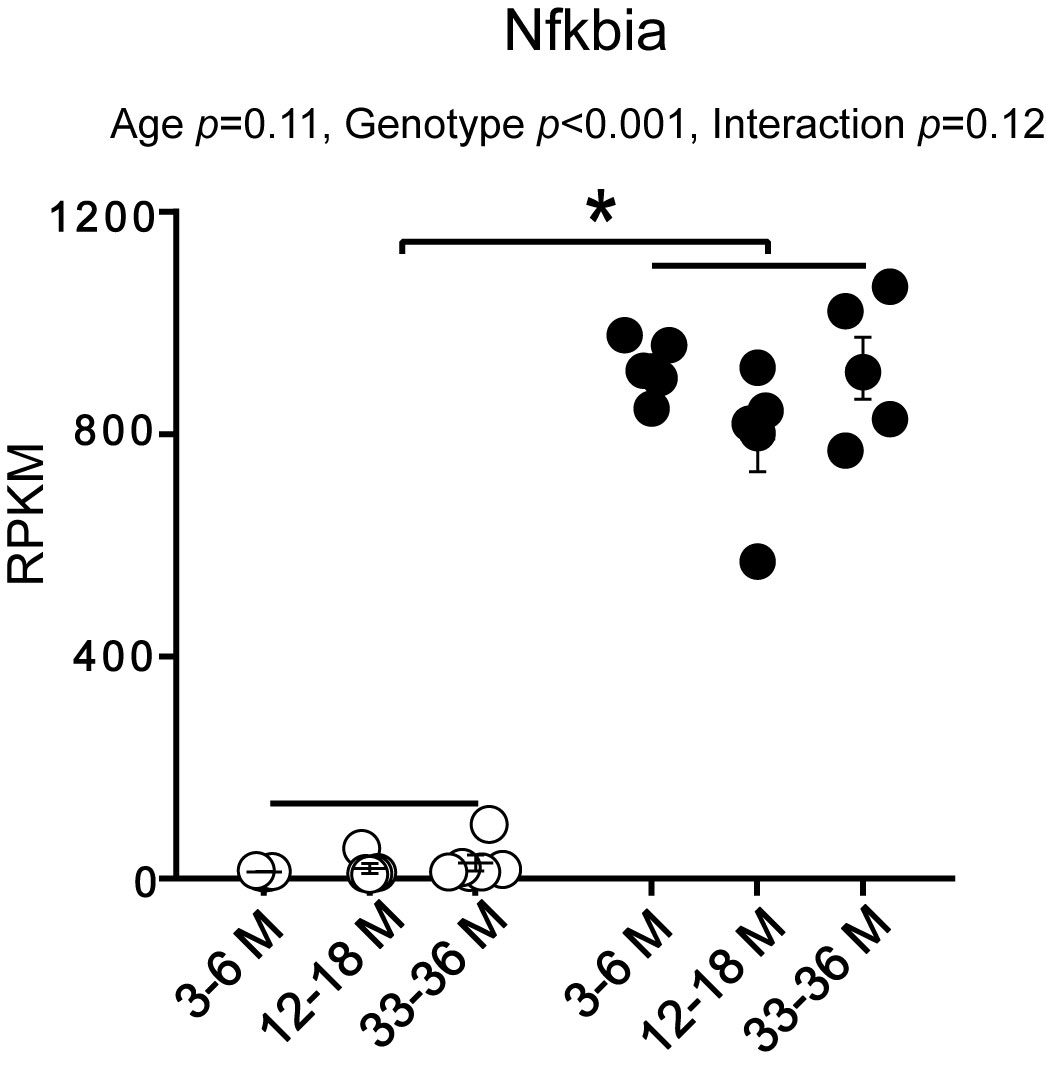


**Supplementary Figure 1**. **mRNA transcript level of *nfkbia* in quadriceps muscle from WT (white) and MISR (black) mice.** n=5-6 per group. Data analyzed by 2-way ANOVA; **P*<0.05 by Tukey’s post-hoc test. All data are means ± SE.

**
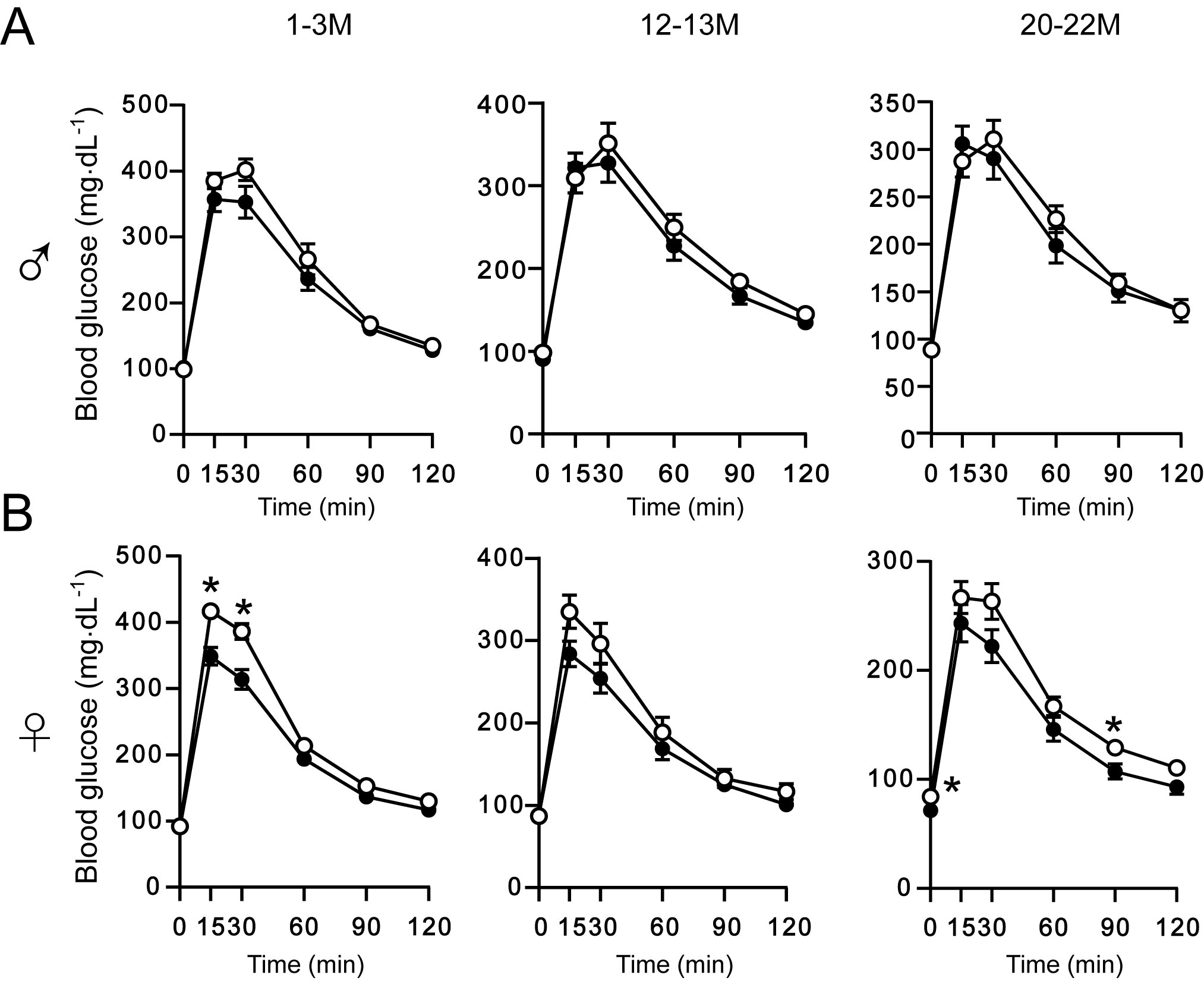
**

**Supplementary Figure 2. Glucose tolerance testing.** Tail blood glucose level was measured before and after i.p. glucose injection in (A) male and (B) female WT (white) and MISR (black) mice of the indicated ages. n=9-10 per group. **P*<0.05 WT vs. MISR from two-tailed unpaired t-test. All data are means ± SE.

A

B

**Supplementary Figure 3.**  **Blood glucose (A) and plasma insulin (B) concentrations during the clamp.** n=4-5 per group. All data are means ± SE.


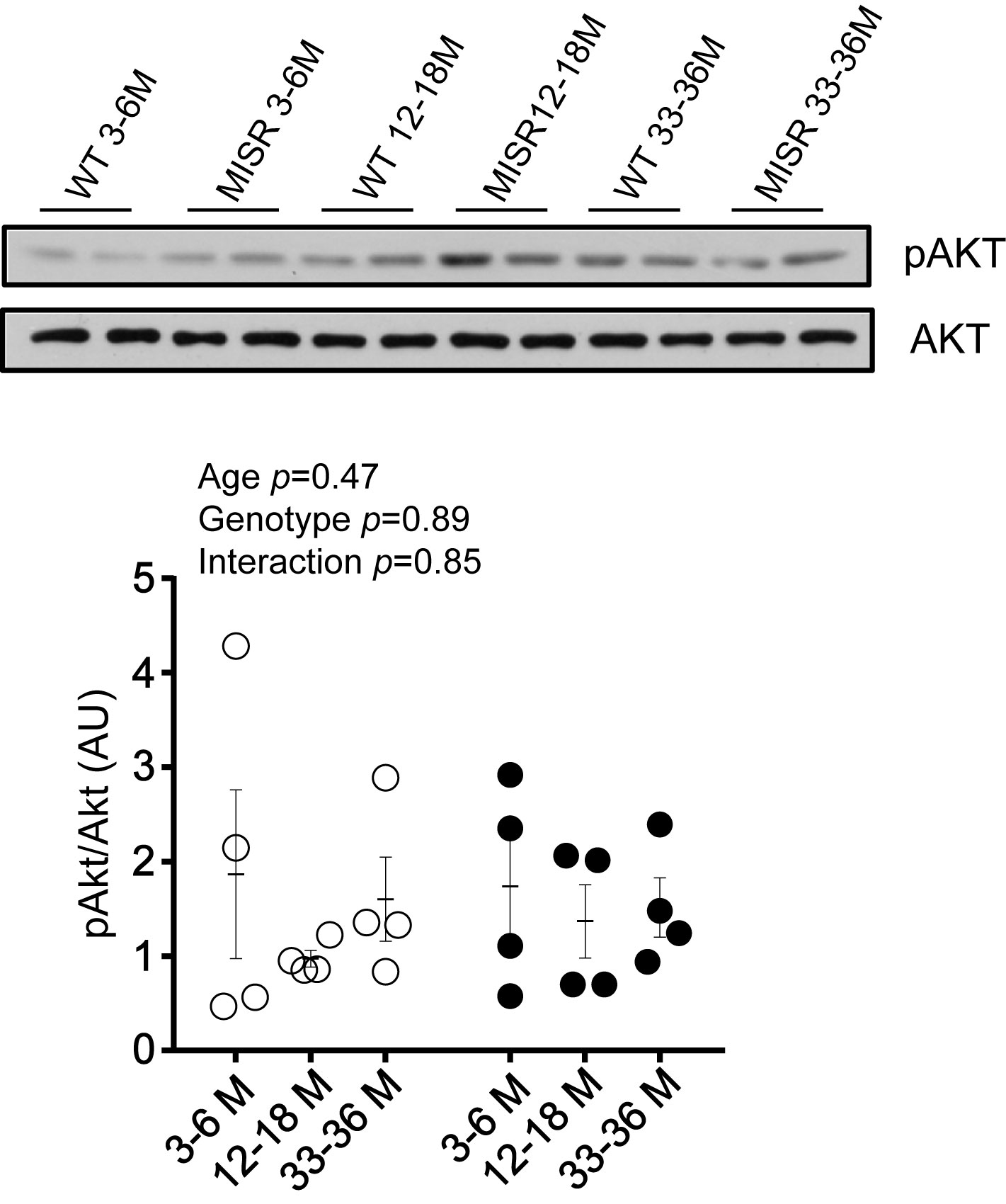


**Supplementary Figure 4. Akt phosphorylation (Ser473) in the baseline (non-insulin stimulated) state assessed by Western blotting in quadriceps muscle**. n=4 per group. Data analyzed by 2-way ANOVA. Data are means ± SE.

B

A

D

C

E

**Supplementary Figure 5. Indirect calorimetry and spontaneous activity measurement.**  (A) Oxygen consumption, (B) carbon dioxide production, (C) respiratory quotient, (D) resting metabolic rate, and (E) spontaneous activity level was measured in 3- (white), 13- (gray), and 35-month-old (black) mice of both genotypes. n=9-20 per group. Data analyzed by 2-way ANOVA. All data are means ± SE.

A

B

**Supplementary Figure 6. Diacylglycerol (DAG) content in quadriceps muscle**. (A) Total and major DAGs. (B) Individual DAG species. n=4-6 per group. §Age effect *P*<0.05 by 2-way ANOVA. **P*<0.05 by Tukey’s post-hoc test. All data are means ± SE.

A

B

**Supplementary Figure 7. Lean mass in male and female mice (Cohort 2).** (A) Lean mass measured in WT (white) and MISR (black) male and (B) female mice of various ages; n=­5-30 in males and n=7-31 in females. Data analyzed by 2-way ANOVA. All data are means ± SE.

A

Adjusted p value <0.05

B


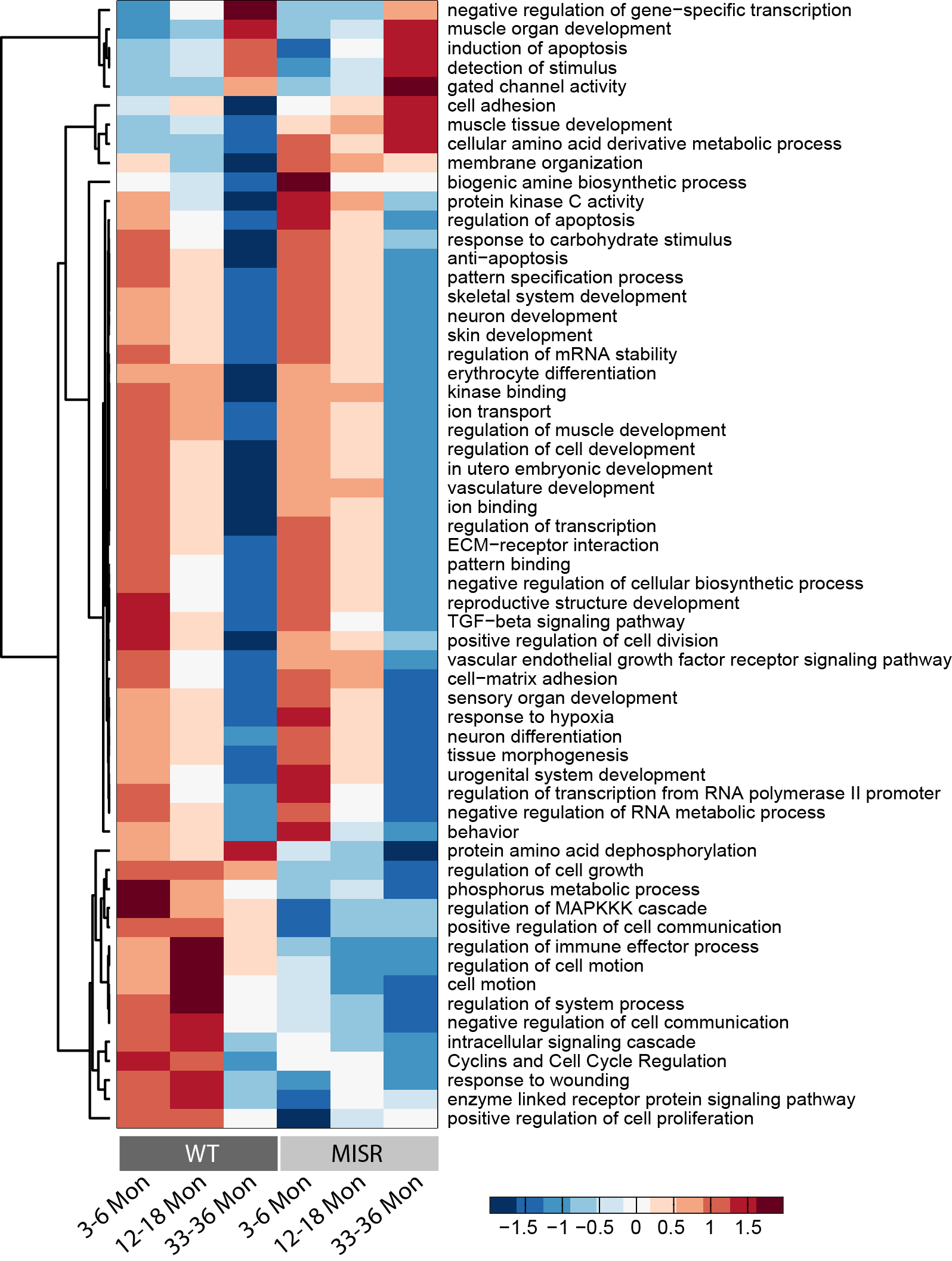


**Supplementary Figure 8.**  Differentially expressed genes selected at higher stringent criterion and the extended gene sets and biology pathways regulated by aging and NFκB suppression. (A) Similar to Figure 4A, number of genes differentially expressed in WT vs. MISR at three different time points with adjusted p-value <0.05 controlled by Benjamini-Hochberg false discovery for multiple tests; and (B) Gene ontology functions and pathway enriched by DAVID analysis. One gene set and pathway per functional cluster was selected for all DAVID functional clusters with enrichment score greater than 1.0, from 5 enrichment analyses (3 DEG lists derived from WT vs. MISR, DEGs from WT 3-6-month-old vs. 33-36-month-old, and DEGs from MISR

3-6-month-old vs. 33-36-month-old groups) for pathways and biological functions differentially regulated by aging and NFκB suppression.


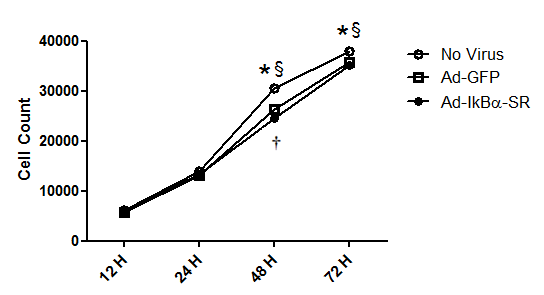


**Supplementary Figure 9. NFκB suppression does not affect myoblast proliferative capacity.** C2C12 myoblasts were seeded at 2,000 cells/well and transduced with Ad-10^7^ IU/well IκBα-SR or Ad-GFP for 12 h before measurement of proliferation over 72 h. **P*<0.05 no virus vs. Ad-GFP; §*P*<0.05 no virus vs. Ad-IκBα-SR; and † *P*<0.05 Ad-GFP vs. Ad-IκBα-SR, from 2-way ANOVA followed by Tukey’s post-hoc test. Data are means ± SE from one experiment done with an n=4 per condition and the experiment was repeated two more times with similar results.


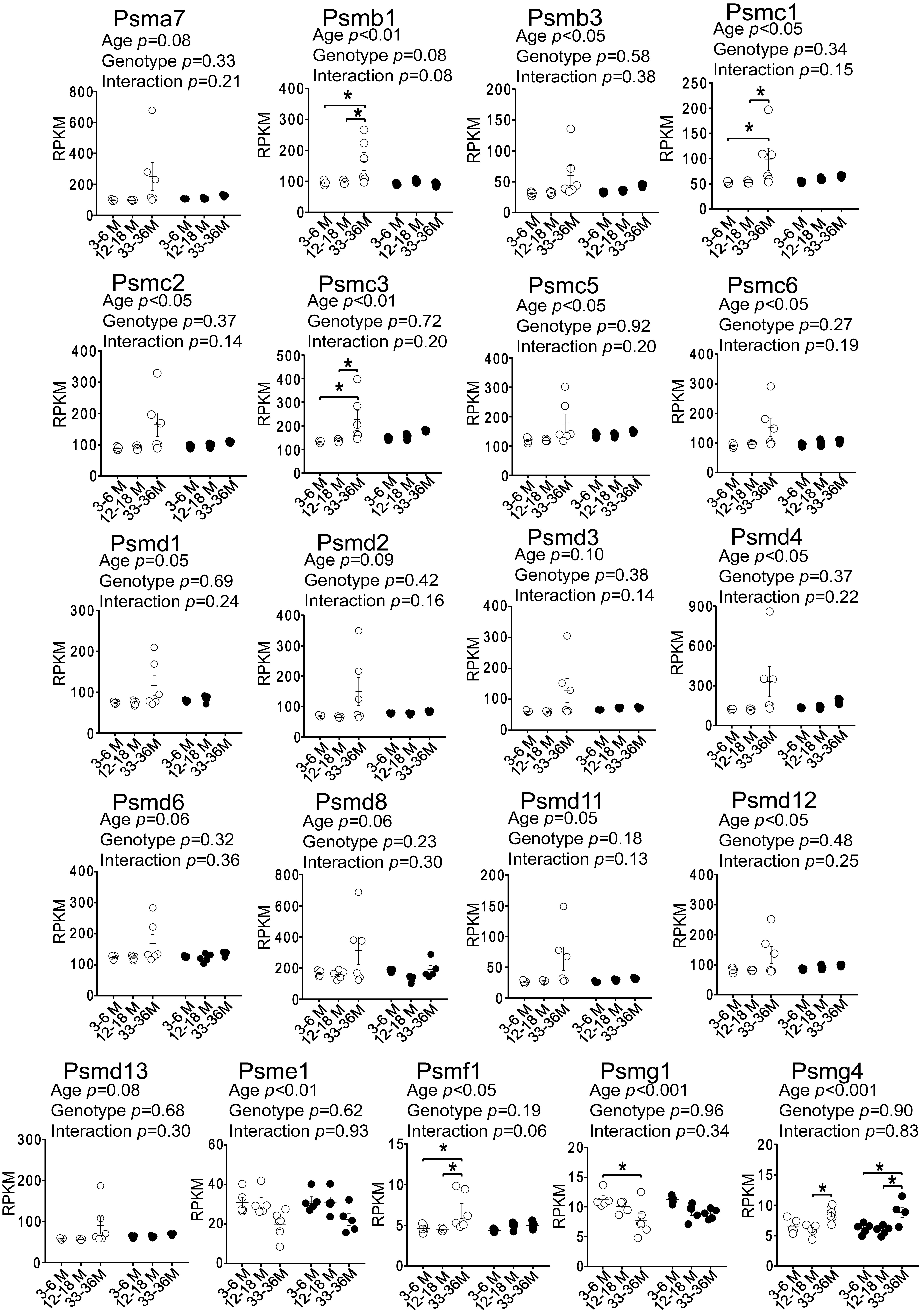


**Supplementary Figure 10. Proteasomal protein gene expression significantly changed with age.** mRNA transcript level in quadriceps muscle from WT (white) and MISR (black) mice. n=5-6 per group. Data analyzed by 2-way ANOVA; **P*<0.05 by Tukey’s post-hoc test. All data are means ± SE.


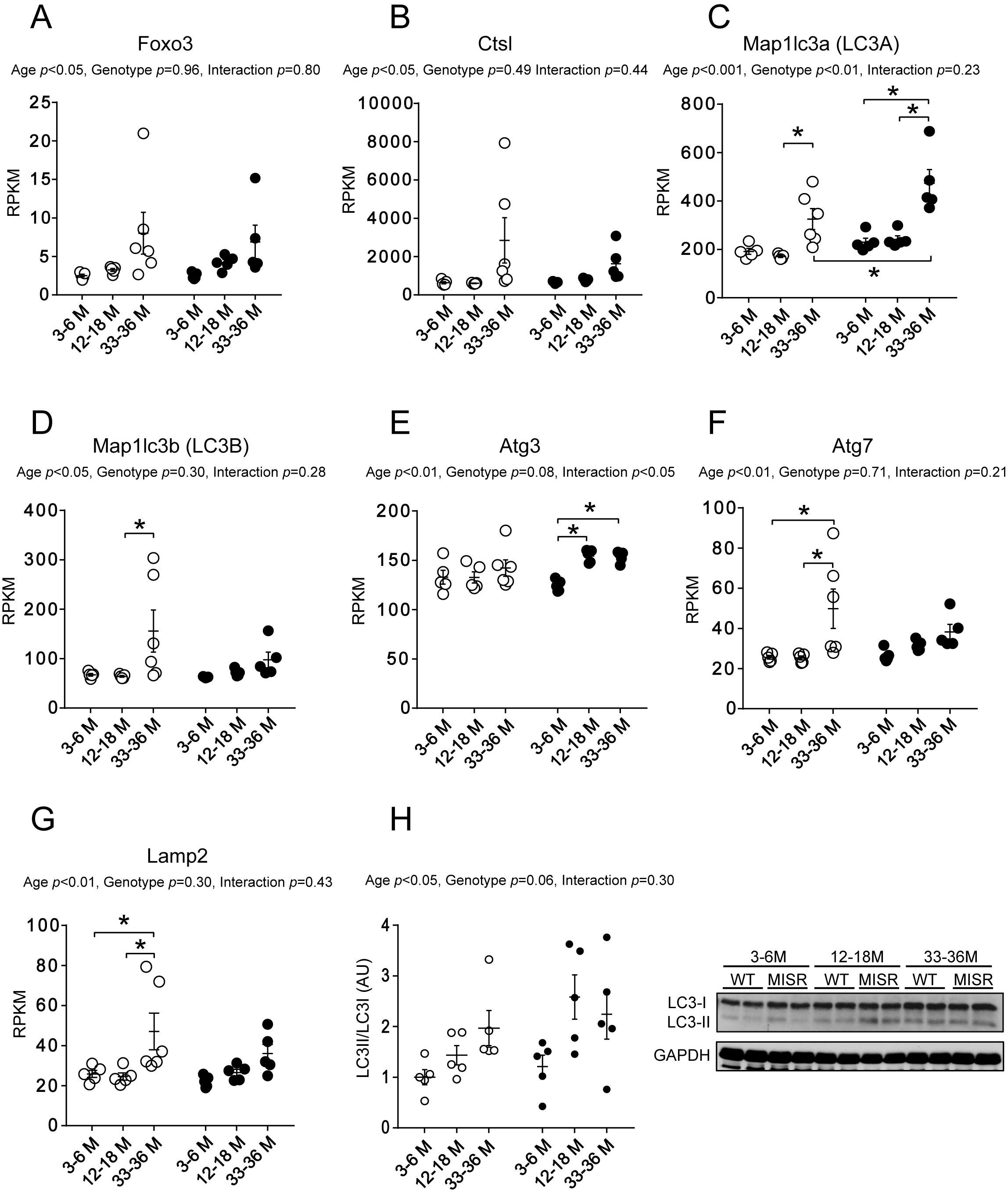


**Supplementary Figure 11. Aging upregulates the autophagy-lysosome pathway.** mRNA expression level for (A) *foxo3*, (B) *ctsl*, (C) *map1lc3a* (LC3A), (D) *map1lc3b* (LC3B), (E) *atg3*, (F) *atg7* and (G) *lamp2*,in quadriceps muscle from WT (white) and MISR (black) overnight fasted mice. n=5-6 per group. (H) LC3-II/LC3-I increases with aging, as determined by Western analysis. n=5 per group. Data analyzed by 2-way ANOVA; **P*<0.05 by Tukey’s post-hoc test. All data are means ± SE.


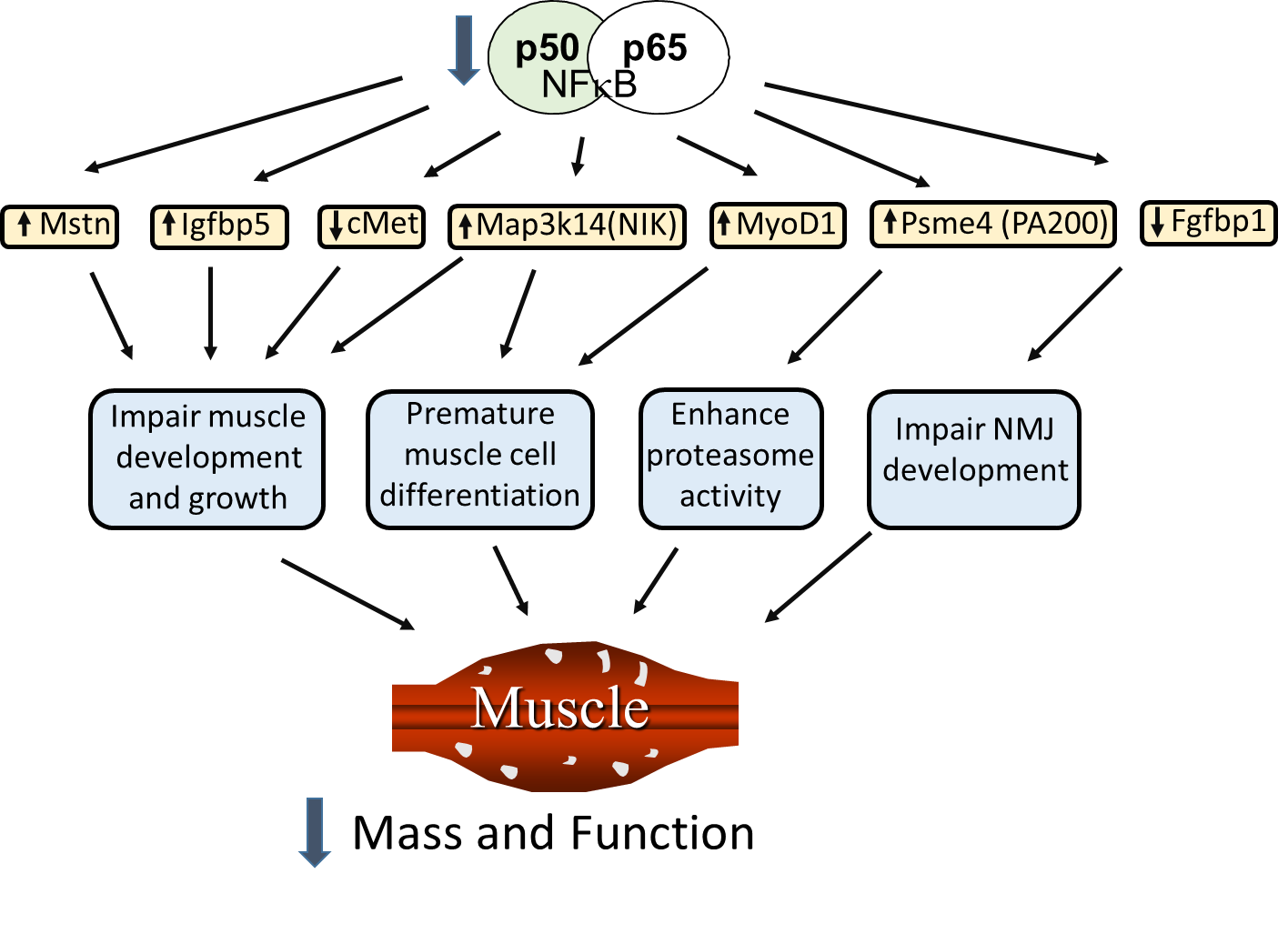


**Supplementary Figure 12. Mechanism underlying reduced muscle mass in MISR mice.** NFκB inhibition leads to reduce muscle mass through various potential mechanisms that involve altered expression of key genes involved in muscle cell differentiation, migration, growth/atrophy and function.


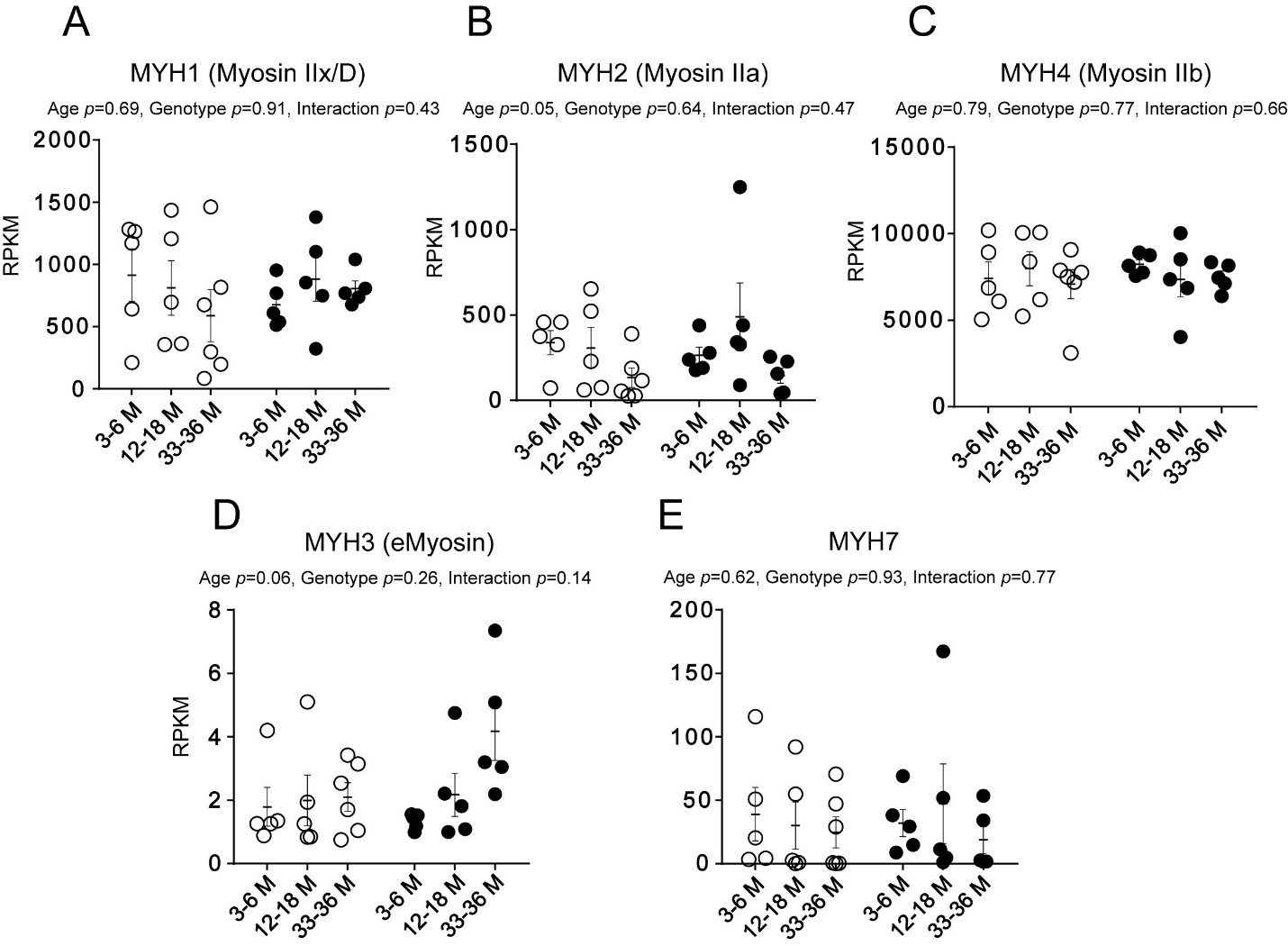


**Supplementary Figure 13. Gene expression levels of myosin heavy chains in quadriceps muscle.** WT (white) and MISR (black). n=5-6 per group. Data analyzed by 2-way ANOVA. Data are means ± SE.


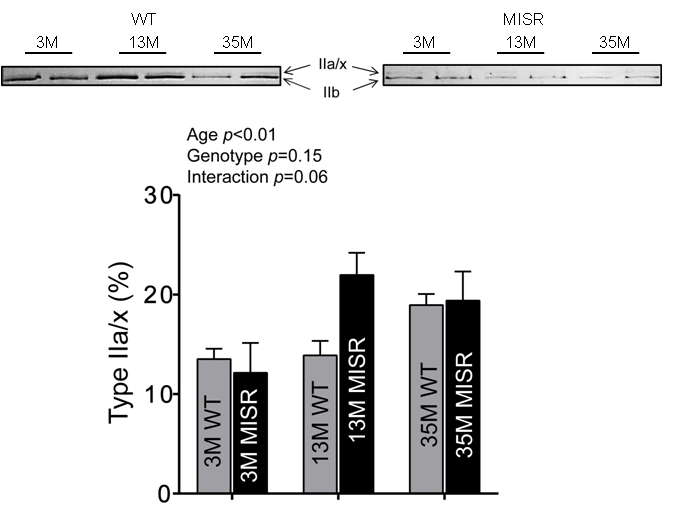


**Supplementary Figure 14. Myosin heavy chain profile.** Myosin heavy chain isoforms from quadriceps muscle were separated by glycerol-SDS-PAGE. Data analyzed by 2-way ANOVA. Data are means ± SE.
