# Supplementary material for: Sustained NFκB inhibition improves insulin sensitivity but is detrimental to muscle health
Source: Aging Cell. 2017 May 29;16(4):847–58. doi: 10.1111/acel.12613 (PMC5506420; doi:10.1111/acel.12613)
Supplement: Supplementary file 4 — Data S1 Procedures and Materials. [file ACEL-16-847-s004.docx]

**Supplemental Information – Procedures and Materials**

**Indirect calorimetry and spontaneous activity.** Following a 24 h acclimation period, oxygen consumption, carbon dioxide production, and resting metabolic rate were measured over a 24 h period using a MARS indirect calorimetry system (Sable Systems International, Las Vegas, NV). Spontaneous activity was assessed in individually-housed mice over 24 h, one light and one dark phase, following a 12 h acclimation period. Mice were placed in clear Plexiglas^®^ cages (40.6 × 22.9 × 14.0 cm) surrounded by a 2.5 cm grid of infrared sensors in the x and y planes, with *ad libitum* access to food and water.

**Nerve conduction assessment.** Sciatic nerve conduction velocity was measured in 3- and 18- month-old male mice as previously described ([Shi *et al.* 2014](#_ENREF_8)). Brieﬂy, proximal ankle electrodes were stimulated, and the response was recorded at the distal foot electrodes placed dorsally over all ﬁve digits. The latency and distance between electrodes were measured, and then the stimulating electrodes were placed at the sciatic notch. The nerve was stimulated again, and the resultant latency was subtracted from the initial ankle-foot latency. This difference was divided between the distance between the notch and ankle to determine velocity.

**Muscle protein content measurements.** Western blotting was performed as described previously ([Liang *et al.* 2013](#_ENREF_6)). For some proteins, their content was measured by capillary electrophoresis using a Wes instrument (Protein Simple, San Jose, CA), following the manufacturer’s protocols and normalizing proteins of interest with total protein content. Primary antibodies used included antibodies against phospho (ser-473) Akt (9271), Akt (9272), α-tubulin (2144s) and GAPDH (2118s) from Cell Signaling (Beverly, MA), NIK (sc-7211), TRAF2 (sc-876) and LC3 (sc-292354) from Santa Cruz Biotechnology (Santa Cruz, CA), fast myosin skeletal heavy chain (M4276) from Sigma-Aldrich (St. Louis, MO), and proteasome 20S α4 subunit (BML-PW8120) from Enzo Life Sciences (Farmingdale, NY).

**Real-time RT-PCR.** *mstn* (Mm01254559__m1) mRNA level was measured by quantitative real-time PCR as described previously (4) on an ABI Prism 7900HT System (Applied Biosystems, Foster City, CA) using TaqMan One-Step RT-PCR Master Mix reagents primer/probes. *polR2j* (Mm00448649_m1) was used as an endogenous control.

**Muscle fiber typing.** Skeletal muscle myosin heavy chain isoforms were separated by glycerol-SDS-PAGE as previously described ([Rockl *et al.* 2007](#_ENREF_7)). Briefly, pulverized quadriceps muscle was homogenized in a lysis buffer (pH 6.5) containing 300 mM KCl, 100 mM KH_2_PO_4_, 50 mM K_2_HPO_4_, and 1 mM EDTA and then centrifuged at 12,000 *g* for 1 min. One μg of muscle proteins were resolved using an SDS-PAGE gel (0.75 mm) consisting of a stacking gel with 4% acrylamide-bis (50:1) and 30% glycerol and a separating gel with 8% acrylamide-bis (50:1) and 30% glycerol. The upper running buffer consisted of 300 mM Tris (base), 450 mM glycine, 0.3% SDS, and 10 mM mercaptoethanol. The lower running buffer consisted of 50 mM Tris (base), 75 mM glycine, and 0.05% SDS. The gels were run on an electrophoresis system at 4°C at a current of 6 mA/gel for 25 h. The gels were fixed for 20 min in 7% acetic acid and 50% methanol, stained for 1 h with Coomasie Blue. Gels were scanned and the band intensity was quantified with ImageQuant TL.

**Lipidomics and metabolomics**. Ceramide and DAG species in quadriceps muscle samples were measured in the Lipidomics Core of the University of South Carolina by high-performance liquid chromatography/mass spectrometry (LC-MS/MS), as previously described ([Bielawski *et al.* 2010](#_ENREF_4)). Relative muscle acylcarnitine, carnitine, and lysine content was measured using LC-MS by Metabolon Inc. (Durham, NC).

**RNA-sequencing and gene expression data analysis.** Total RNAs, isolated using TRIzol reagent (Invitrogen), were prepared from frozen quadriceps muscles, each with at least 5 replicates. RNA quality was assessed by Bioanalyzer (Agilent Technologies) and mRNA was isolated by oligo-dT purification and fragmented using divalent cations under elevated temperature. cDNA fragment libraries were synthesized following the TruSeq mRNA-seq Library Preparation protocol (Illumina, San Diego, CA), and sequenced with an Illumina HiSeq 2000 system at the Greehey Children’s Cancer Research Institute Genome Sequencing Facility (GSF), employing a 50bp single-read sequencing protocol. On average, we obtained ~45 million reads per sample.

After sequencing, short sequence reads were first aligned to mouse genome (UCSC mm9) using TopHat aligner ([Trapnell *et al.* 2009](#_ENREF_9)), then reads aligned to known transcripts were counted using HTSeq ([Anders *et al.* 2015](#_ENREF_2)). Expression abundance of each gene was quantitated both by the total reads aligned to the gene (read count) and by RPKM (read per kilobase of transcript per million reads mapped). Differential gene expression analysis was performed using DESeq ([Anders & Huber 2010](#_ENREF_1)) to obtain fold-change, *p*-value, and adjusted *p*-value by Benjamini-Hochberg correction for multiple tests ([Benjamini & Hochberg 1995](#_ENREF_3)). We selected differentially expressed genes based on the following criteria: 1) fold-change > 1.5, 2) adjusted *p*-value < 0.05, and 3) baseMean (mean read counts) > 10, or otherwise specified. Functional assessment of differentially expressed genes was performed using Database for Annotation, Visualization and Integrated Discovery (DAVID, <http://david.abcc.ncifcrf.gov/>) ([Huang da *et al.* 2009](#_ENREF_5)) and Ingenuity pathway analysis (IPA, Qiagen, <http://www.ingenuity.com>).

To generate functional gene set and/or pathway based heatmap, we first selected functional enriched terms or pathways from DAVID and IPA analysis (after removing *nfkbia*, which is the gene overexpressed in MISR mice). For a given gene set with *M* genes, we calculated its summarized expression level as $Y=\sum_{i=1}^{M} a_{i}x_{i}$, where *x_i_* is the ith gene’s log-transformed expression level, and parameter $a_{i}\in[-1, 1]$ is the sign of the Pearson correlation of gene **x** to the average profile of the given gene set. Note that *x_i_* is a vector of gene expression across 6 conditions (WT at 3-6, 12-18 and 33-36 months of age, and MISR at 3-6, 12-18 and 33-36 months of age). We then perform *z*-transformation of *Y* to represent the expression change across 6 conditions. *z* > 0 is coded to be magenta color, and *z* < 0 light blue color in the final heatmap.

**REFERENCES**

Anders S, Huber W (2010). Differential expression analysis for sequence count data. *Genome biology*. **11**, R106.

Anders S, Pyl PT, Huber W (2015). HTSeq--a Python framework to work with high-throughput sequencing data. *Bioinformatics*. **31**, 166-169.

Benjamini Y, Hochberg Y (1995). Controlling the False Discovery Rate: A Practical and Powerful Approach to Multiple Testing. *Journal of the Royal Statistical Society. Series B (Methodological)*. **57**, 289-300.

Bielawski J, Pierce JS, Snider J, Rembiesa B, Szulc ZM, Bielawska A (2010). Sphingolipid analysis by high performance liquid chromatography-tandem mass spectrometry (HPLC-MS/MS). *Advances in experimental medicine and biology*. **688**, 46-59.

Huang da W, Sherman BT, Lempicki RA (2009). Systematic and integrative analysis of large gene lists using DAVID bioinformatics resources. *Nature protocols*. **4**, 44-57.

Liang H, Hussey SE, Sanchez-Avila A, Tantiwong P, Musi N (2013). Effect of lipopolysaccharide on inflammation and insulin action in human muscle. *PloS one*. **8**, e63983.

Rockl KS, Hirshman MF, Brandauer J, Fujii N, Witters LA, Goodyear LJ (2007). Skeletal muscle adaptation to exercise training: AMP-activated protein kinase mediates muscle fiber type shift. *Diabetes*. **56**, 2062-2069.

Shi Y, Ivannikov MV, Walsh ME, Liu Y, Zhang Y, Jaramillo CA, Macleod GT, Van Remmen H (2014). The lack of CuZnSOD leads to impaired neurotransmitter release, neuromuscular junction destabilization and reduced muscle strength in mice. *PloS one*. **9**, e100834.

Trapnell C, Pachter L, Salzberg SL (2009). TopHat: discovering splice junctions with RNA-Seq. *Bioinformatics*. **25**, 1105-1111.
